# The burden of disease in Russia from 1980 to 2016: a systematic analysis for the Global Burden of Disease Study 2016

**DOI:** 10.1016/S0140-6736(18)31485-5

**Published:** 2018-09-29

**Authors:** GBD 2016 Russia Collaborators, Vladimir I Starodubov, Laurie B Marczak, Elena Varavikova, Boris Bikbov, Sergey P Ermakov, Julia Gall, Scott D Glenn, Max Griswold, Bulat Idrisov, Michael Kravchenko, Dmitry Lioznov, Enrique Loyola, Ivo Rakovac, Sergey K Vladimirov, Vasiliy Vlassov, Christopher J L Murray, Mohsen Naghavi

## Abstract

**Background:**

Over the past few decades, social and economic changes have had substantial effects on health and wellbeing in Russia. We aimed to use data from the Global Burden of Diseases, Injuries, and Risk Factors Study 2016 (GBD 2016) to evaluate trends in mortality, causes of death, years lived with disability (YLDs), years of life lost (YLLs), disability-adjusted life-years (DALYs), and associated risk factors in Russia from 1980 to 2016.

**Methods:**

We estimated all-cause mortality by use of a multistage modelling process that synthesised data from vital registration systems, surveys, and censuses. A composite measure of health loss due to both fatal and non-fatal disease burden (DALYs) was calculated as the sum of YLLs and YLDs for each age, sex, year, and location. Health progress was evaluated in comparison with patterns of change in similar countries by use of the Socio-demographic Index that was developed for GBD 2016.

**Findings:**

Following rapid decreases in life expectancy after the collapse of the Soviet Union, life expectancy at birth in Russia improved between 2006 and 2016. The all-cause mortality rate decreased by 16·6% (95% uncertainty interval 9·4–33·8) between 1980 and 2016. This overall decrease encompasses the cycles of sharp increases and plateaus in mortality that occurred before 2005. Child mortality decreased by 57·5% (53·5–61·1) between 2000 and 2016. However, compared with countries at similar Socio-demographic Index levels, rates of mortality and disability in Russia remain high and life expectancy is low. Russian men have a disproportionate burden of disease relative to women. In 2016, 59·2% (55·3–62·6) of mortality in men aged 15–49 years and 46·8% (44·5–49·5) of mortality in women were attributable to behavioural risk factors, including alcohol use, drug use, and smoking.

**Interpretation:**

Trends in mortality in Russia from 1980 to 2016 might be related to complicated patterns of behavioural risk factors associated with economic and social change, to shifts in disease burden, and to changes in the capacity of and access to health care. Ongoing mortality and disability from causes and risks amenable to health-care interventions and behaviour modifications present opportunities to continue to improve the wellbeing of Russian citizens.

**Funding:**

Bill & Melinda Gates Foundation.

## Introduction

After episodes of disruption and interim improvements in life expectancy at birth in Russia and other former Soviet republics in the 1990s, the decade of 2006 to 2016 was characterised by reductions in mortality from many causes of death and a steady improvement in life expectancy. These mortality reductions have been substantial over the past decade, in many cases reversing the trends in increasing mortality that were initiated by the collapse of the Soviet Union. These reductions reflect changes in social and economic conditions, better health policies, and improved outcomes of many diseases.

The purpose of our study was to describe the trends in life expectancy, the causes of mortality, and the contribution of major risk factors to mortality and morbidity in Russia compared with those in selected comparator countries from 1990 to 2016. Russia has expertise and data on demographic and epidemiological trends in the country and, although some of these patterns have been presented elsewhere,^1^, ^2^, ^3^ the Global Burden of Diseases, Injuries, and Risk Factors Study 2016 (GBD 2016) contributes a comprehensive estimation of morbidity and mortality by cause, age, and sex, and allows benchmarking of these metrics in Russia compared with data from other countries. These analyses support the development of policy priorities that are based on the most up to date and accurate information about health, disability, and mortality.

## Methods

We focused on the data and analyses from the GBD 2016 study that quantified mortality, years of life lost (YLLs), years lived with disability (YLDs), and life expectancy at birth in Russia and comparator countries. Additional results from our study and from GBD 2016 can be explored with online data visualisation tools and downloaded by use of a results query tool. We reported all rates as age-standardised rates derived from world population standards that were developed for the GBD study, and each point estimate includes 95% uncertainty intervals (UIs).

**Research in context**

**Evidence before this study**

We searched for articles published in PubMed on or before Sept 1, 2017, with the search terms “Russia”, “burden”, “mortality”, and “health”; relevant papers were translated as necessary. Cause-specific mortality and morbidity in Russia have been detailed by previous studies, many of which are available in Russian language publications or from government repositories. Our expertise allowed us to identify and translate information from the Russian Federal State Statistics Service, and to access Russian language assessments of life expectancy and mortality trends, tuberculosis, and the alcohol-related burden of disease. These analyses generally evaluated a single cause or groups of causes, often focused on specific subpopulations by age or sex, and restricted the analytical frame to Russia.

**Added value of this study**

To our knowledge, this is the first study of Russia to comprehensively estimate the frequency of, causes of, and risk factors associated with mortality and morbidity by sex and age over a long period of time in Russia. By use of standardised methods to control for data quality and completeness, we facilitated benchmarking of health progress in Russia against its regional and socioeconomic peers. To achieve this goal, we evaluated 333 causes of death and disability and 84 risk factors by age, sex, and year between 1980 and 2016.

**Implications of all the available evidence**

Although the rate of all-cause mortality in Russia remains greater than for the other countries in the highest quintile of socioeconomic development, life expectancy at birth has now returned to the levels observed before the economic and social events of the 1990s. Regional similarities in persisting patterns of increased mortality associated with behavioural risk factors, particularly for men of working age, appear related to similarities in political and social contexts that have developed since the collapse of the Soviet Union. This work represents a first step in understanding the patterns and trends in disease burden across Russia by age and by sex. The complexity of Russian geography supports the use of this comparative evaluation on a national scale as a base onto which more detailed subnational investigations can be added.

Estimates incorporated data from vital registration systems, surveys, and censuses; all data sources used in this analysis can be explored in detail with the online data source tool. Details of the development and implementation of GBD methods for addressing variation in data quality are published elsewhere.^4^ The cause of death database developed for GBD 2016 was used to generate cause-specific estimates of mortality rate by location, year, sex, and age for 264 causes of death.^4^ The Cause of Death Ensemble model and Bayesian meta-regression were used to generate estimates of mortality and morbidity by cause for each combination of location, year, age, and sex. Adult mortality and mortality of children younger than 5 years were modelled separately. In conjunction with established GBD study methods, YLLs were calculated by multiplying cause-specific deaths within each age group by the normative standard life expectancy at each age. Total YLDs were calculated after adjusting for disease severity, exclusivity, and comorbidity. All-cause and cause-specific disability-adjusted life-years (DALYs)—a composite measure of health loss due to both fatal and non-fatal disease burden—were calculated as the sum of YLLs and YLDs for each combination of age, sex, year, and location. The GBD study employs a comparative risk assessment framework to quantify a set of behavioural, environmental and occupational, and metabolic risks by use of evidence rules for the inclusion of risk-outcome pairs in the analysis. The attributable burden of deaths or YLLs for a risk-outcome pair was calculated as the total number of deaths or YLLs for that outcome, multiplied by the calculated fraction of cases in a population that were attributable to a specific exposure. Additional details of the GBD 2016 comparative risk assessment and attribution methods are published elsewhere.^5^, ^6^

Changes in life expectancy and causes of death in Russia were compared with patterns of change in seven comparison countries, which were selected to include those with the longest shared borders with Russia (Finland, Ukraine, Kazakhstan, China, and Japan) to provide regional comparisons, and included Germany and the USA to represent other advanced economies. GBD 2016 uses the Socio-demographic Index (SDI) to compare health progress between locations.^4^ Because of the greater availability and reliability of data on all-cause mortality compared with cause-specific mortality, all-cause mortality and life expectancy estimates are reported for 1980–2016, but cause of death analyses are limited to 1990–2016.

### Role of the funding source

The funder of the study had no role in study design, data collection, data analysis, data interpretation, or writing of the report. The corresponding author had full access to all the data in the study and had final responsibility to submit for publication.

## Results

The lowest life expectancy in Russia during 1980–2016 was estimated to be in 1994, when life expectancy for both sexes combined was 63·7 (95% UI 62·5–64·9) years (appendix). In 2016, male life expectancy at birth in Russia was 65·4 (60·8–70·7) years, but life expectancy at birth for women reached an historical high of 76·2 (71·4–80·7) years. This disparity in life expectancy between men and women decreased slightly from 1980 to 2016, from 11·6 years in 1980 to 10·9 years in 2016. However, this disparity between the sexes remains the greatest of any country worldwide.

All-cause, age-standardised rates of DALYs in Russia, Kazakhstan, and Ukraine increased sharply and then plateaued between 1990 and 1995. DALYs in Russia subsequently decreased from the peak rate of 53 140·5 (95% UI 48 656·8–58 111·1) DALYs per 100 000 people in 1994 to 43 056 (34 476·2–53 258·6) DALYs per 100 000 people in 2016, representing an annualised decline of 3·1% (1·1–4·9; appendix). Similarly, the all-cause mortality rate in Russia in 2016 decreased by 16·6% (9·4–33·8) relative to 1980 levels (appendix). This overall decrease in mortality rate was largely driven by changes after 2005. Similar to all-cause rates of DALYS, all-cause mortality was dominated by sharp increases from 1991 to 1994, a plateau, and then a 26·3% (5·2–43·3) decrease from 2005 to 2016. Overall rates of mortality in Russia in 2016 were between 1·4 and 2·7 times greater than rates for the comparator countries that are not former Soviet republics.

**Figure 1:**
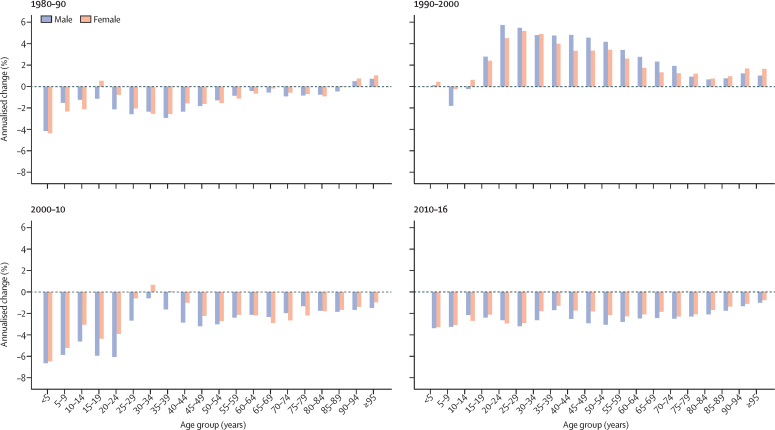
Annualised change in all-cause mortality, stratified by age group and sex in 1980–90, 1990–2000, 2000–10, and 2010–16

Annualised rates of change (AROC) of all-cause mortality rate were negative for most age groups in most time intervals, and the largest decreases were observed in children younger than 5 years (figure 1). The notable exception to this finding was in the decade following the collapse of the Soviet Union (1990–2000), when AROC were positive (reflective of the higher mortality rates) for all age groups except for children aged 5–9 years and boys aged 10–14 years. AROC in this period were notably high for men of working age, remaining above 4% for age groups between 20 and 54 years and for women between 20 and 39 years. In 2010–16, negative AROC were shown for all age groups; the maximum AROC in this period was a negative rate of change of 3·4% (95% UI 1·7–4·9) for males younger than 5 years. Most of the observed decreases in mortality in children younger than 5 years occurred between 2000 and 2016, when mortality decreased by 57·5% (53·5–61·1; appendix). Between 1980 and 2016, child mortality decreased by 71·4% (68·7–73·8) in Russia.

**Figure 2:**
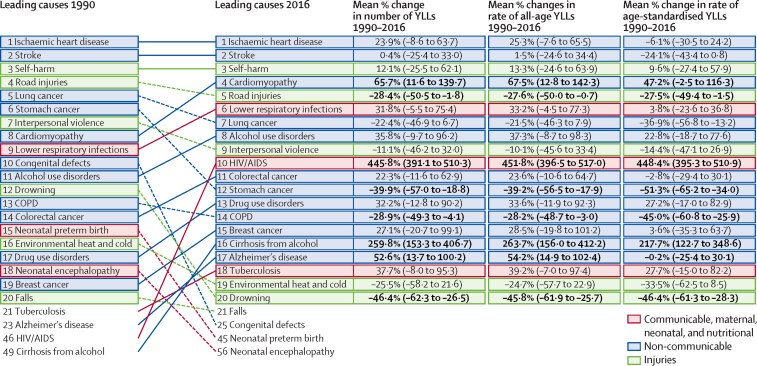
Relative rank of the leading 20 causes of YLLs in 1990 and 2016, the change in number of YLLs, and age-standardised change in number of YLLs between 1990 and 2016 Data are ranked by Global Burden of Disease Level 3 causes. Bold text indicates statistical significance. Causes are connected by arrows between time periods; solid lines are increases in rank and dashed lines are decreases in rank. COPD=chronic obstructive pulmonary disease. YLL=years of life lost.

Ischaemic heart disease and cerebrovascular disease were leading causes of YLLs in Russia in 1990 and in 2016 (figure 2). Self-harm, the third leading cause of YLLs in Russia in 2016, was also unchanged in rank order from 1990. However, there have been changes in the relative rank of the other top 20 causes of YLLs. Cardiomyopathy (predominantly from alcohol), lower respiratory infections, alcohol use disorders, HIV/AIDS, colorectal cancer, drug use disorders, breast cancer, cirrhosis from alcohol, Alzheimer's disease, and tuberculosis all increased in their relative contribution to YLLs by more than two rank positions between 1990 and 2016. Among the leading causes of YLLs, the largest decreases in age-standardised rates of YLLs between 1990 and 2016 were reported for stomach cancer (51·3%, 95% UI 34·0–65·2), drowning (46·4%, 28·3–61·3), COPD (45·0%, 25·9–60·8), and lung cancer (36·9%, 13·2–56·8). Between 1990 and 2016, the greatest increases in age-standardised rates of YLLs were observed for HIV/AIDS (448·4 years, 395·3–510·9) and cirrhosis and other chronic liver diseases due to alcohol use (217·7 years, 122·7–348·6). The leading 20 sources of YLDs in Russia in 2016 were mostly non-communicable diseases, including alcohol use disorder and drug use disorder, except for falls (third leading source of YLDs), road injuries (14th), and mechanical forces (20th; appendix).

**Figure 3:**
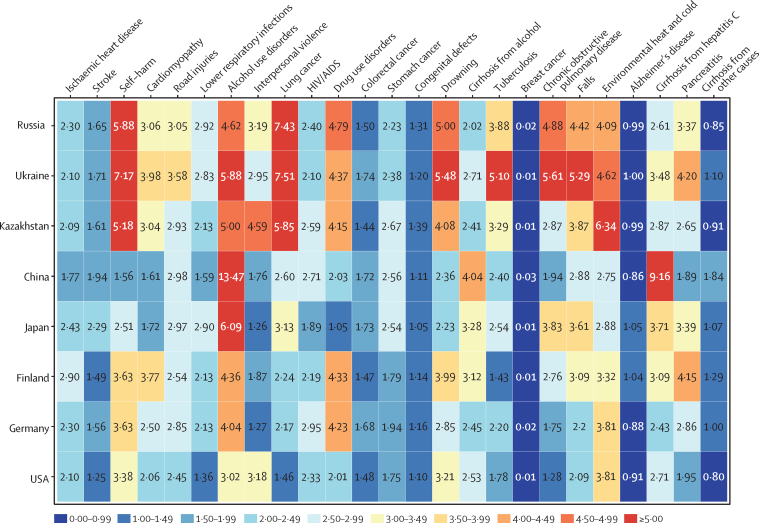
Ratio of male to female age-standardised rates of years of life lost in 2016 among Russia and comparator countries for the 20 leading causes of death in Russia

In Russia in 2016, YLL rates from the leading 20 causes were higher in men than in women for all causes except for breast cancer, cirrhosis from causes other than alcohol or hepatitis C, and Alzheimer's disease (figure 3). The ratio of YLLs by men to YLLs by women was skewed most strongly towards men for lung cancer, in whom the rate of YLLs in 2016 was 1197·6 (95% UI 806·1–1635·9) YLLs per 100 000 people, which was 7·4 times the equivalent rate for women. This ratio was similarly skewed towards men in Ukraine and Kazakhstan; however, in other comparator countries, YLLs for lung cancer were not as strongly skewed by men. In Russia, rates of YLLs in men from self-harm, drug use disorders, alcohol use disorders, COPD, environmental heat and cold, and falls were all more than four times the equivalent rates in women in 2016. Other than COPD in Kazakhstan, where YLLs in men were 2·9 times greater than those in women, these sex-specific patterns of YLLs were broadly similar to those in Ukraine and Kazakhstan. With a few exceptions, disparities between YLLs by men and women were greater in Russia, Ukraine, and Kazakhstan than other comparator countries.

**Figure 4:**
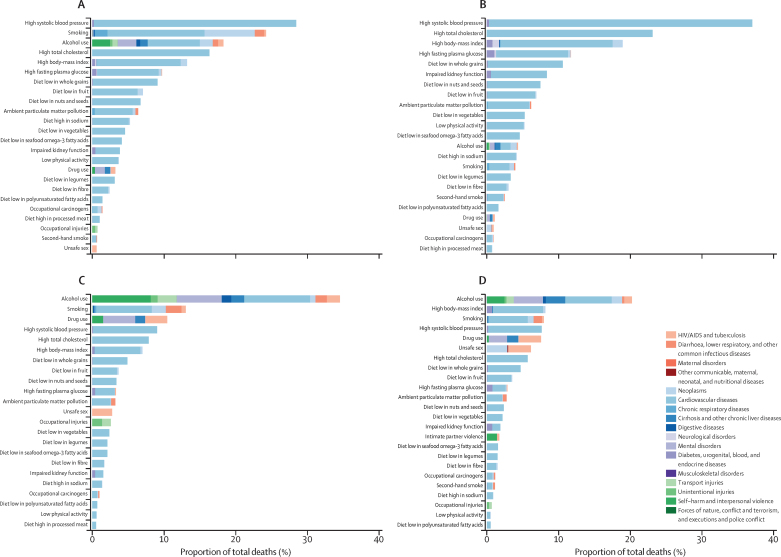
Proportion of deaths at level 3 of the Global Burden of Diseases Study hierarchy attributable to each risk factor in Russia, 2016 Data are expressed as percentage of total deaths for (A) males of all ages; (B) females of all ages; (C) males aged 15–49 years; and (D) females aged 15–49 years. Risks attributable to less than 1% of deaths are omitted.

In 2016, 53·4% (95% UI 48·7–57·8) of deaths in Russia were attributable to behavioural risk factors, 48·5% (45·1–51·7) were attributable to metabolic risks, and 8·2% (7·2–9·3) were attributable to environmental and occupational risks (appendix). In both sexes (of all ages), high systolic blood pressure was the leading risk factor (figure 4) and was responsible for 32·7% (28·4–36·7) of deaths in Russia in 2016. These deaths were mostly due to the resulting cardiovascular disease. For males, smoking was the second most common risk factor and was responsible for 24·1% (21·7–26·5) of deaths, followed in attributable burden by alcohol use, which was responsible for 18·2% (13·6–22·6) of deaths and was a factor in several leading causes of death in Russia (figure 4). For females, high total cholesterol was the second most common risk factor and was associated with 23·2% (16·4–30·7) of deaths, and high body-mass index was the third most common risk factor and was associated with 19·0% (12·4–25·4) of deaths (figure 4).

Behavioural risk factors were particularly relevant to the mortality of adults aged 15–49 years and were associated with 59·2% (95% UI 55·3–62·6) of deaths in males and 46·8% (44·5–49·5) of deaths in females (figure 4) in 2016. For males in this age group, alcohol use as a risk factor was responsible for 34·4% (29·5–38·6) of deaths in 2016 versus 20·1% (17·1–23·4) of deaths in females. In comparison, the highest attributions of deaths to alcohol use among comparator countries for males in this age group were in Ukraine, where alcohol was associated with 33·1% (28·5–37·9) of deaths; Kazakhstan, where it is was associated with 25·4% (19·7–31·2) of deaths; and Finland, where it was associated with 21·5% (16·0–27·4) of deaths (appendix). Of deaths in males aged 15–49 years in Russia in 2016, cardiovascular disease attributed to alcohol use comprised 9·1% (7·5–11·0) of deaths, alcohol-attributed transport injury comprised 2·6% (1·6–3·9) of deaths, alcohol-attributed self-harm and violence comprised 8·1% (5·6–11·0) of deaths, and mental and substance use disorders attributed to alcohol comprised 6·1% (5·3–7·0) of deaths (figure 4).

**Figure 5:**
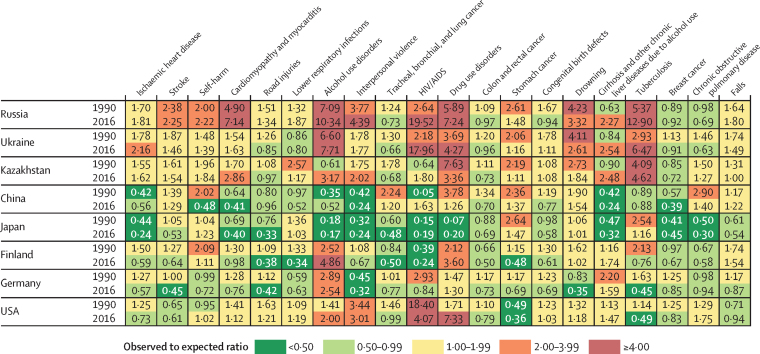
Ratio of observed to expected age-standardised rates of years of life lost for both sexes in 1990 and 2016 among Russia and comparator countries for the 20 leading causes of death in Russia

In Russia in 2016, increases in the ratio of observed to expected YLLs (based on SDI level) exceeded 4:1 for cardiomyopathy (observed to expected ratio 7·14), alcohol use disorders (10·34), interpersonal violence (4·39), HIV/AIDS (19·52), drug use disorders (7·24), and tuberculosis (12·90; figure 5). In comparator countries in 2016, this ratio exceeded 4:1 for alcohol use disorders (7·71), HIV/AIDS (17·96), drug use disorders (4·27), and tuberculosis (6·47) in Ukraine; tuberculosis (4·62) in Kazakhstan; alcohol use disorders (4·86) in Finland; and for HIV/AIDS (4·07) and drug use disorders (7·33) in the USA. By contrast, the ratio of observed to expected YLLs in Russia in 2016 had decreased relative to that of 1990 for stroke; road injuries; tracheal, bronchial, and lung cancer; colon and rectal cancer; stomach cancer; congenital birth defects; drowning; and COPD.

## Discussion

As with other former Soviet republics, cycles of decreasing and increasing life expectancy and mortality rates occurred in Russia in the years following the dissolution of the Soviet Union. During the 1990s and 2000s, social and economic turmoil produced an acute mortality crisis in former Soviet republics. Several potential causes for this mortality crisis and its subsequent echoes have been suggested.^7^, ^8^, ^9^ Stress associated with the transition from Soviet economies to capitalism has been linked with an increased risk of circulatory diseases, which are exacerbated by increased alcohol consumption as a behavioural response to stress.^10^ This interpretation is supported by the rapid recovery of life expectancy in the Baltic states during the late 1990s^11^ while life expectancy in Russia continued to be affected by political and social upheavals, such as the economic crisis of 1998. Behavioural risk factors such as alcohol use and smoking, and metabolic risk factors including high systolic blood pressure, high total cholesterol, and high body-mass index continue to contribute strongly to the burden of disease in Russia. The loss of effective public health interventions through successive changes to health policies probably interacted with cultural and economic factors to magnify the effects of socioeconomic transition on mortality.

Economic and social turmoil often increase the mortality of infants and the elderly,^12^ yet we did not observe this pattern in Russia. Mortality rates decreased for older adults and for those younger than 20 years, particularly children younger than 5 years since 2000, in conjunction with continuing declines in mortality from both maternal and neonatal disorders. Probable explanations for these patterns include the success of the acute care system in reducing deaths from infectious disease and deaths occurring in infancy,^13^ the previous success of the Soviet health system in many of its efforts to reduce communicable diseases and set up comprehensive childhood vaccination programmes, and improvements in care for many aspects of cardiovascular disease that have lessened adverse outcomes for older patients.^13^, ^14^

In contrast to decreases in mortality for older adults and children younger than 5 years, we found that mortality rates in Russian adults increased from 1990 to 2000, particularly in men aged 20–34 years. One of the most notable characteristics of the mortality patterns in Russia in the past 36 years is the high mortality rates in men who are of working age. Rapid increases in death rates at working age in Russia, particularly in men, have been linked to socioeconomic upheaval following the dissolution of the Soviet Union in 1989 and the economic crisis in 2008.^14^ Across the leading causes of premature mortality in Russia, the burden for men remains greater than that for women, most notably in the case of lung cancer and alcohol-related conditions (which have mirrored the cycling patterns of mortality of the post-Soviet era) but also for self-harm, drug use, COPD, and falls. The prevalence of smoking in women in Russia is low, particularly relative to its prevalence in men,^15^ which is reflected in the extent of the disparity in burden between the sexes from associated diseases; for example, in 2016, mortality from lung cancer (rates of which were 12 times higher in men) and cardiovascular diseases (rates of which were six times higher in men than women) were both higher in men. In Russia, as in most countries, women tend to live longer than men, albeit with higher levels of disability in both absolute and relative terms.

When ranked by SDI, Russia is 33rd globally, which is within the highest quintile of scores among countries. Despite the potential suggested by this ranking, premature mortality in 2016 from several leading causes (such as tuberculosis, alcohol use disorders, and HIV/AIDS) was more than ten times greater than expected based on SDI alone. Clear direct links exist between alcohol and causes of death such as alcohol poisoning, liver cirrhosis, alcoholic cardiomyopathy, and stroke.^16^ Alcohol use is also a behavioural component of other causes of death; many types of injuries and violence are associated with inebriation^17^, ^18^ and connections between alcohol use, drug use, and infectious disease such as tuberculosis and HIV/AIDS are well known.^19^ We have found increases in mortality from HIV/AIDs in Russia between 1990 and 2016 that were relatively unaffected by the cycles of mortality correlated with economic upheaval that were observed for other causes of death. The continuing increase in mortality associated with HIV/AIDS in Russia has been attributed to unsafe sex^20^ and to drug use,^21^ with both causes exacerbated by the cultural context of alcohol use.^19^ Certain populations, such as individuals with tuberculosis, could also be more vulnerable to negative health outcomes caused by the interaction of physiological and behavioural components of heavy alcohol use.^22^ Prison populations, in particular, serve as a nexus for the transmission of HIV/AIDS, viral hepatitis, and tuberculosis; the prevalence of multidrug resistant tuberculosis in prisons of post-Soviet states is estimated to be 16 times the worldwide prevalence.^23^ Migrant worker populations are another vulnerable population because they can lack access to health care.^24^

In a widely cited retrospective case-control study^25^ in Russia (that was supported by a subsequent prospective observational study^26^), Zaridze and colleagues reported that, of those aged 15–54 years, 59% of deaths in men and 33% of deaths in women were attributable to alcohol. By contrast, our study estimated that, of those aged 15–49 years, 34·4% (29·5–38·6) of deaths in males and 20·1% (17·1–23·4) of deaths in females were attributable to alcohol. There are several probable reasons for the disparity in these estimates. Participants in the retrospective case-control study were recruited from urban locations in Siberia where heavy drinking might be more prevalent than elsewhere in Russia. Additionally, the study by Zaridze and colleagues^25^ used an implausibly high level of relative risk for injuries, contributing to the higher estimated attributable deaths than our study. GBD 2016 estimated the relative risk across outcomes from alcohol use from a meta-analysis of prospective cohorts that encompassed 336 000 individuals to derive continuous relative risks and included systematic controls for likely confounding variables.^6^

Russia (together with other former Soviet republics, particularly Ukraine) continues to be among the countries with the highest alcohol-related mortality globally; the contribution of drug use disorders to mortality in Russia also remains at high levels. After the introduction of restrictive alcohol policies in the 1980s, reductions in mortality from causes attributable to alcohol were observed, and the more recent decreases in alcohol-related mortality can be similarly correlated with public health measures that were introduced in the mid-2000s.^27^ Our results document substantial trend reversals in rates of mortality from alcoholic cardiomyopathy, self-harm, alcohol use and drug use disorders, and cirrhosis due to alcohol in the last decade. These improvements have not fully ameliorated the mortality peaks that occurred in the mid-1990s and mid-2000s, however, and despite these recent decreases, mortality rates attributable to alcohol and drug use remain higher for Russia than for other countries at comparable levels of development. The substantial role of alcohol and drug abuse in premature mortality in Russia is both avoidable and demonstrably responsive to national policy.

In contrast to successes that have improved the trends in morbidity and mortality related to alcohol use, the prevalence of smoking in Russia remains high and increases in smoking have been noted for women.^15^ Simultaneously, taxes on tobacco in Russia remain among the lowest in the world.^28^ Ross and colleagues^28^ found that the higher taxes collected in countries such as Poland and Ukraine since the early 1990s have contributed to higher rates of smoking cessation in those countries compared with Russia. As with alcohol use, policies are available that could lead to decreased smoking prevalence and a reduction in the overall burden of disease; such policies include increased taxes on tobacco, education on health outcomes, improved access to cessation treatments, and restrictions on public consumption. Legislative measures^29^ that started in 2010 hold promise for reducing Russia's tobacco burden. Additionally, the government has been recognised by WHO for implementing substantive policies towards monitoring tobacco use and prevention.^30^

Limitations of our study include those described for the whole GBD 2016 study.^4^ Despite robust vital registration data, information on morbidity and health outcomes associated with risk factors are comparatively limited for Russia; specifically, inpatient and outpatient hospital data from Russia were not available for the GBD 2016 study. Because of the uncertainty associated with the evidence used to quantify the risks associated with different patterns of alcohol use (such as binge drinking), we assumed uniform consumption of alcohol across the year; greater measurement of the effects of episodic consumption (both in prospective studies and surveys that evaluate consumption) are required before this effect can be considered for incorporation into the GBD study. Additionally, GBD 2016 estimated mortality and morbidity in Russia at the national level. Due to its large geography and population, health patterns in Russia have substantial diversity that would be better captured by subnational estimation.

Despite substantial improvements across many maternal and neonatal sources of mortality between 1980 and 2016, some communicable diseases, particularly the nexus of HIV/AIDS and tuberculosis and their interaction with substance use disorders, have shown less improvement. The association between mortality, alcohol, and social stress is notable for its enduring role in trends in the burden of disease in Russia. Mortality from causes amenable to health care or behavioural modification, such as hypertension, cerebrovascular disease, smoking, and alcohol-related disease, remains high, indicating a substantial scope for future improvements in health in Russia.

Correspondence to: Prof Mohsen Naghavi, Institute of Health Metrics and Evaluation, University of Washington, Seattle, WA 98121, USA **nagham@uw.edu**
