# Supplementary material for: The burden of disease in Russia from 1980 to 2016: a systematic analysis for the Global Burden of Disease Study 2016
Source: Lancet. 2018 Sep 29;392(10153):1138–46. doi: 10.1016/S0140-6736(18)31485-5 (PMC6172188; doi:10.1016/S0140-6736(18)31485-5)
Supplement: Supplementary appendix [file mmc1.pdf]

THE LANCET

Supplementary appendix

This appendix formed part of the original submission and has been peer reviewed. We post it as supplied by the authors.

Supplement to: GBD 2016 Russia Collaborators. The burden of disease in Russia from 1980 to 2016: a systematic analysis for the Global Burden of Disease Study 2016. *Lancet* 2018; published online Aug 30. http://dx.doi.org/10.1016/S0140-6736(18)31485-5.

Trends in life expectancy at birth for Russia and comparator countries, both sexes combined, 1980−2016

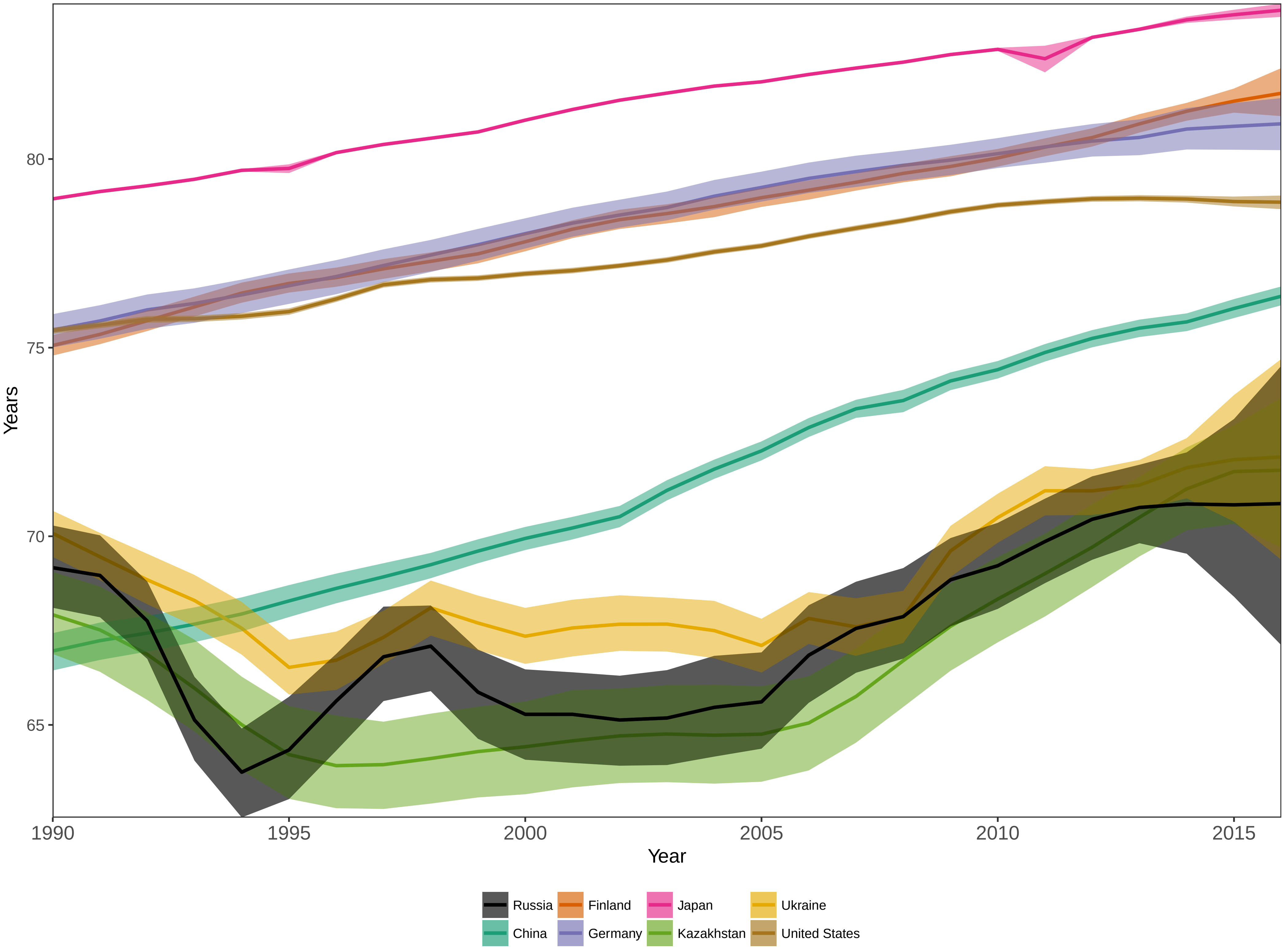

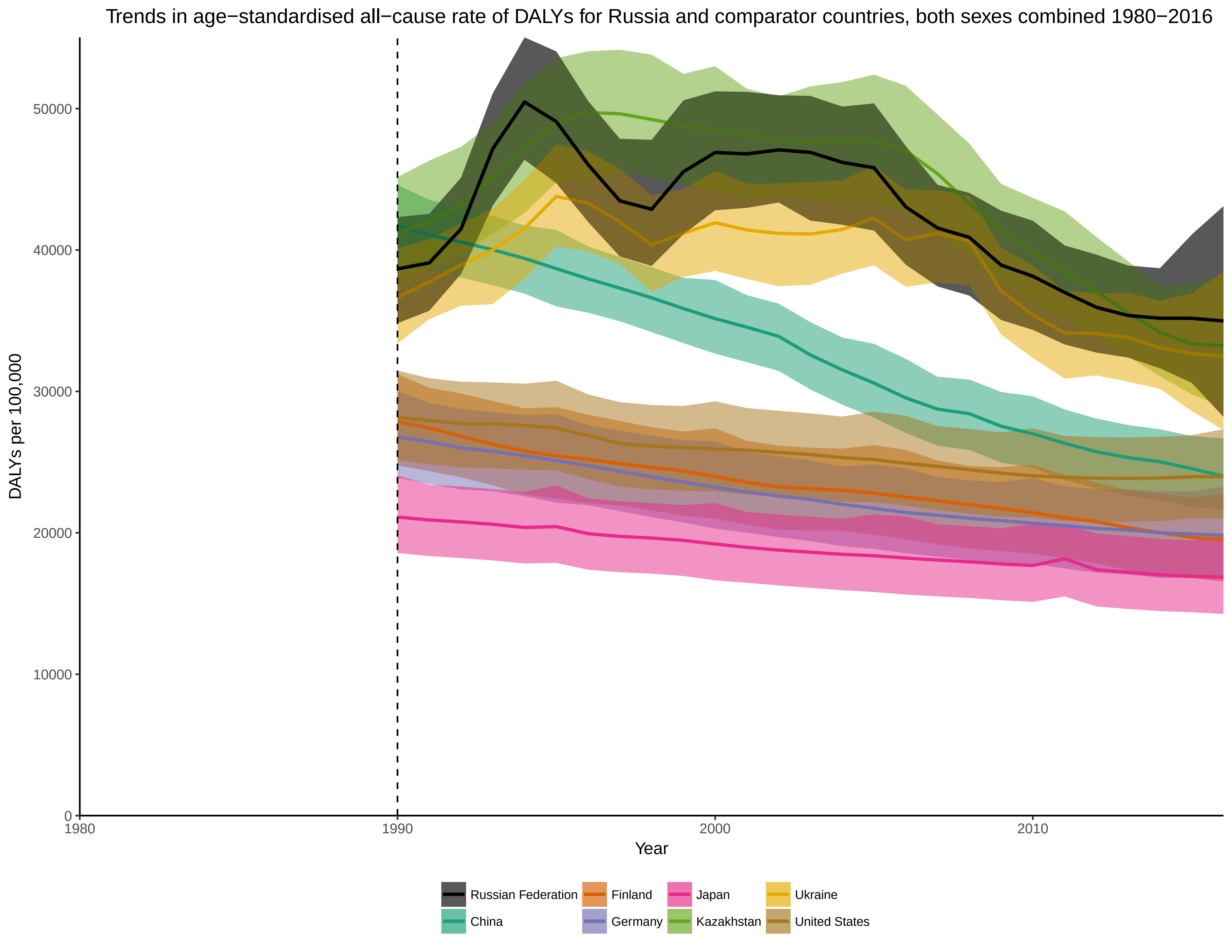
Trends in age−standardised all−cause rate of DALYs for Russia and comparator countries, both sexes combined 1980−2016
DALYs per 100,000
50000
40000
30000
20000
10000
0
1980
1990
2000
2010
Year
Russian Federation
Finland
Japan
Ukraine
China
Germany
Kazakhstan
United States

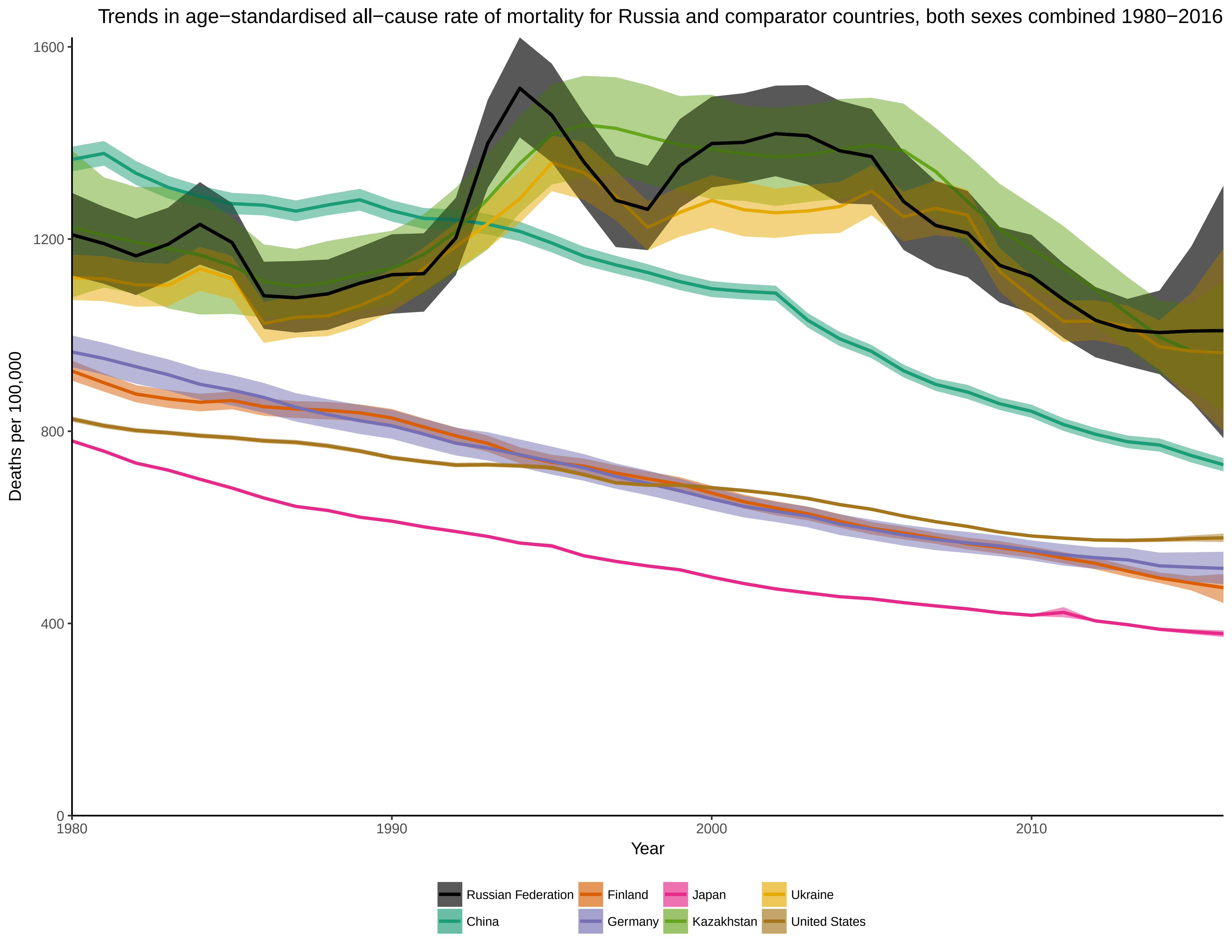

Trends in age−standardised all−cause rate of mortality for Russia and comparator countries, both sexes combined 1980−2016
Deaths per 100,000
1600
1200
800
400
0
1980
1990
2000
2010
Year
Russian Federation
China
Finland
Germany
Japan
Kazakhstan
Ukraine
United States

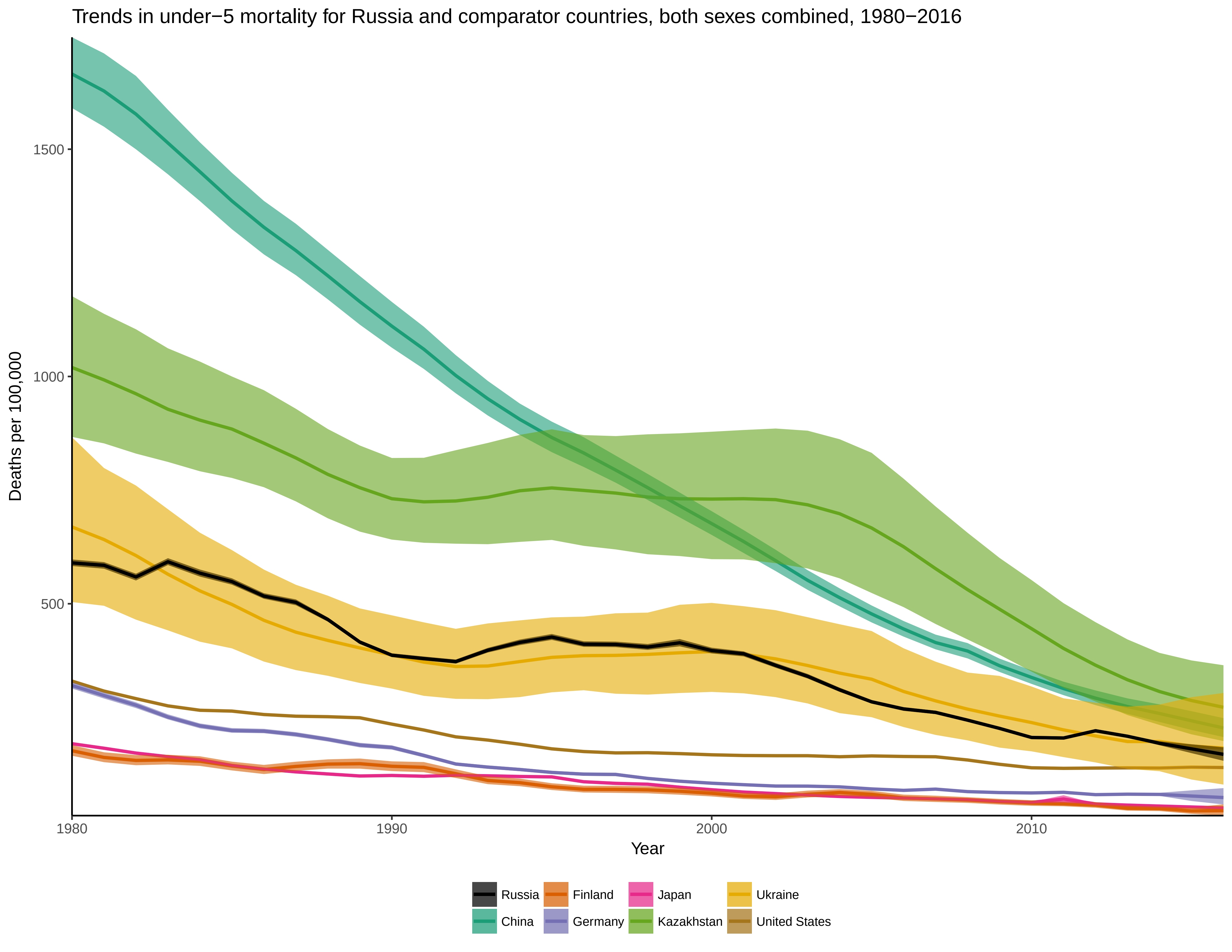
Trends in under−5 mortality for Russia and comparator countries, both sexes combined, 1980−2016
Deaths per 100,000
1500
1000
500
1980
1990
2000
2010
Year
Russia
Finland
Japan
Ukraine
China
Germany
Kazakhstan
United States

Top 20 level 3 causes of YLDs for 1990 and 2016, Russia Both sexes

Leading causes 1990	Leading causes 2016	Mean % change number of YLDs 1990-2016	Mean % change all-age YLD rate 1990-2016	Mean % change age-standardized YLD rate 1990-2016
1 Low back & neck pain	1 Low back & neck pain	**5.8% (1.1 to 10.3%)**	**6.9% (2.2 to 11.5%)**	**-8.2% (-11.9 to -4.5%)**
2 Sense organ diseases	2 Sense organ diseases	**20.0% (17.9 to 22.6%)**	**21.3% (19.2 to 23.9%)**	**-2.4% (-3.9 to -0.8%)**
3 Migraine	3 Falls	**10.8% (7.4 to 13.8%)**	**12.1% (8.6 to 15.1%)**	**-3.9% (-6.8 to -1.3%)**
4 Falls	4 Migraine	**3.8% (0.5 to 7.4%)**	**4.9% (1.6 to 8.5%)**	0.2% (-2.8 to 3.3%)
5 Depressive disorders	5 Depressive disorders	**5.7% (0.6 to 11.3%)**	**6.8% (1.7 to 12.5%)**	-3.8% (-8.0 to 0.6%)
6 Skin diseases	6 Skin diseases	**-4.9% (-7.1 to -2.8%)**	**-3.8% (-6.1 to -1.7%)**	1.9% (-0.1 to 4.2%)
7 Stroke	7 Stroke	**18.0% (8.0 to 27.3%)**	**19.3% (9.2 to 28.7%)**	**-10.6% (-18.0 to -3.7%)**
8 Alcohol use disorders	8 Alcohol use disorders	4.3% (-4.5 to 13.8%)	5.5% (-3.5 to 15.0%)	-0.7% (-9.1 to 8.2%)
9 Drug use disorders	9 Oral disorders	**18.3% (15.4 to 21.7%)**	**19.6% (16.7 to 23.0%)**	0.2% (-1.6 to 2.2%)
10 Oral disorders	10 Osteoarthritis	**35.7% (31.0 to 40.5%)**	**37.2% (32.4 to 42.0%)**	**8.1% (4.4 to 11.8%)**
11 Diabetes	11 Diabetes	**26.4% (13.7 to 39.6%)**	**27.8% (15.0 to 41.1%)**	2.1% (-8.0 to 12.6%)
12 Osteoarthritis	12 Drug use disorders	-6.3% (-14.9 to 2.6%)	-5.3% (-14.0 to 3.7%)	-8.2% (-17.0 to 0.8%)
13 Anxiety disorders	13 Anxiety disorders	1.6% (-4.7 to 8.3%)	2.7% (-3.7 to 9.5%)	-0.2% (-6.4 to 6.2%)
14 Road injuries	14 Road injuries	**12.1% (8.8 to 15.5%)**	**13.4% (9.9 to 16.7%)**	-1.7% (-4.4 to 0.9%)
15 Asthma	15 Ischemic heart disease	**29.7% (23.9 to 35.2%)**	**31.1% (25.2 to 36.6%)**	-0.6% (-4.8 to 3.5%)
16 Gynecological diseases	16 Asthma	**-9.7% (-14.6 to -4.9%)**	**-8.7% (-13.7 to -3.8%)**	**-9.4% (-14.4 to -4.8%)**
17 Congenital defects	17 Gynecological diseases	0.2% (-3.7 to 4.0%)	1.3% (-2.6 to 5.1%)	-2.3% (-6.1 to 1.6%)
18 Mechanical forces	18 Other mental & substance	**9.3% (4.2 to 14.2%)**	**10.5% (5.4 to 15.5%)**	0.4% (-4.2 to 5.1%)
19 Upper respiratory infect	19 Upper respiratory infect	**-6.6% (-10.9 to -2.4%)**	**-5.5% (-9.9 to -1.3%)**	-0.1% (-3.9 to 3.9%)
20 Ischemic heart disease	20 Mechanical forces	**-10.6% (-14.6 to -7.0%)**	**-9.6% (-13.7 to -6.0%)**	**-19.4% (-22.8 to -16.3%)**
21 Other mental & substance	22 Congenital defects			

Legend:

Communicable, maternal, neonatal and nutritional

Non-communicable

Injuries

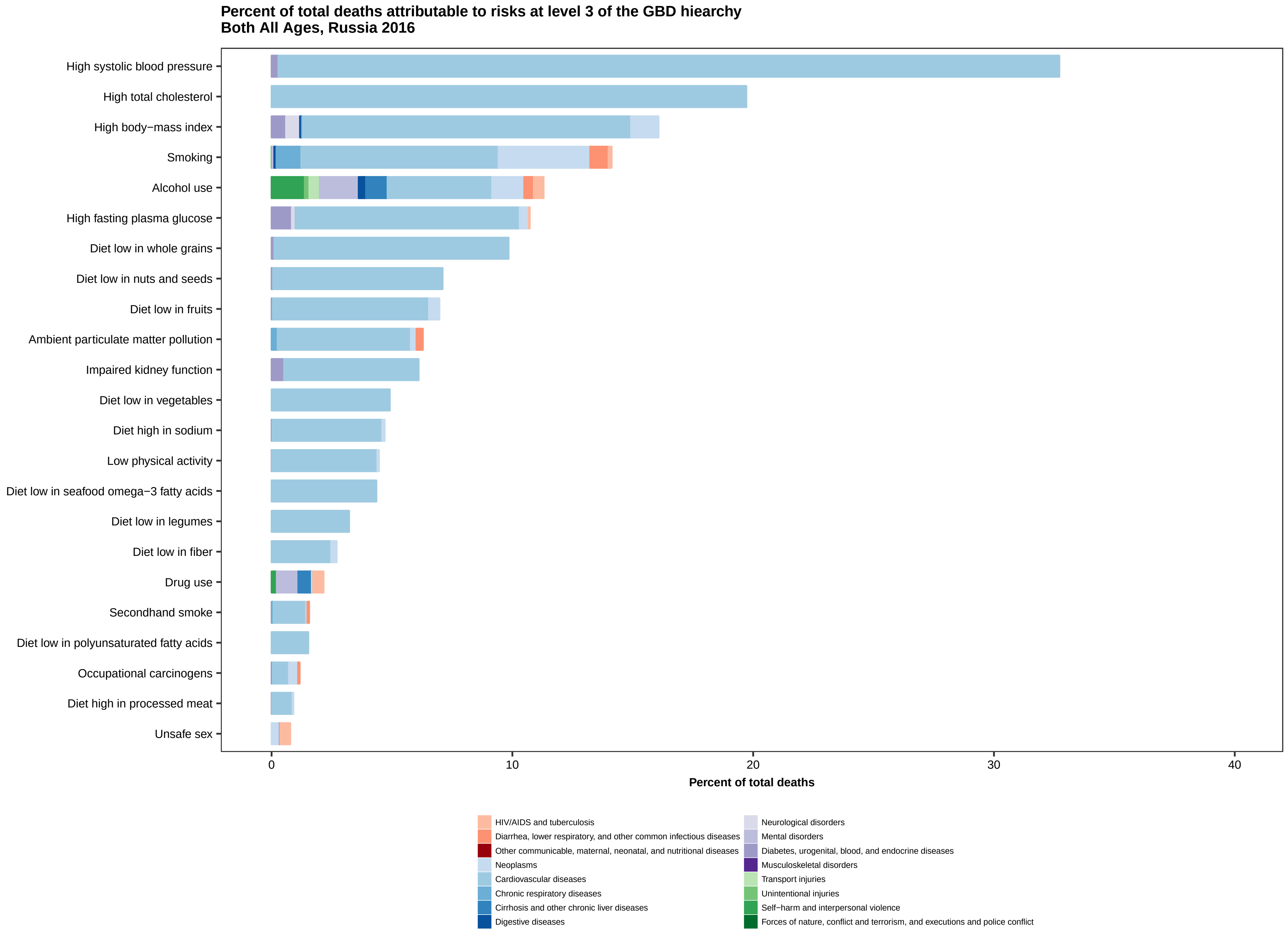

Risks attributable to less than 1% of total deathss omitted

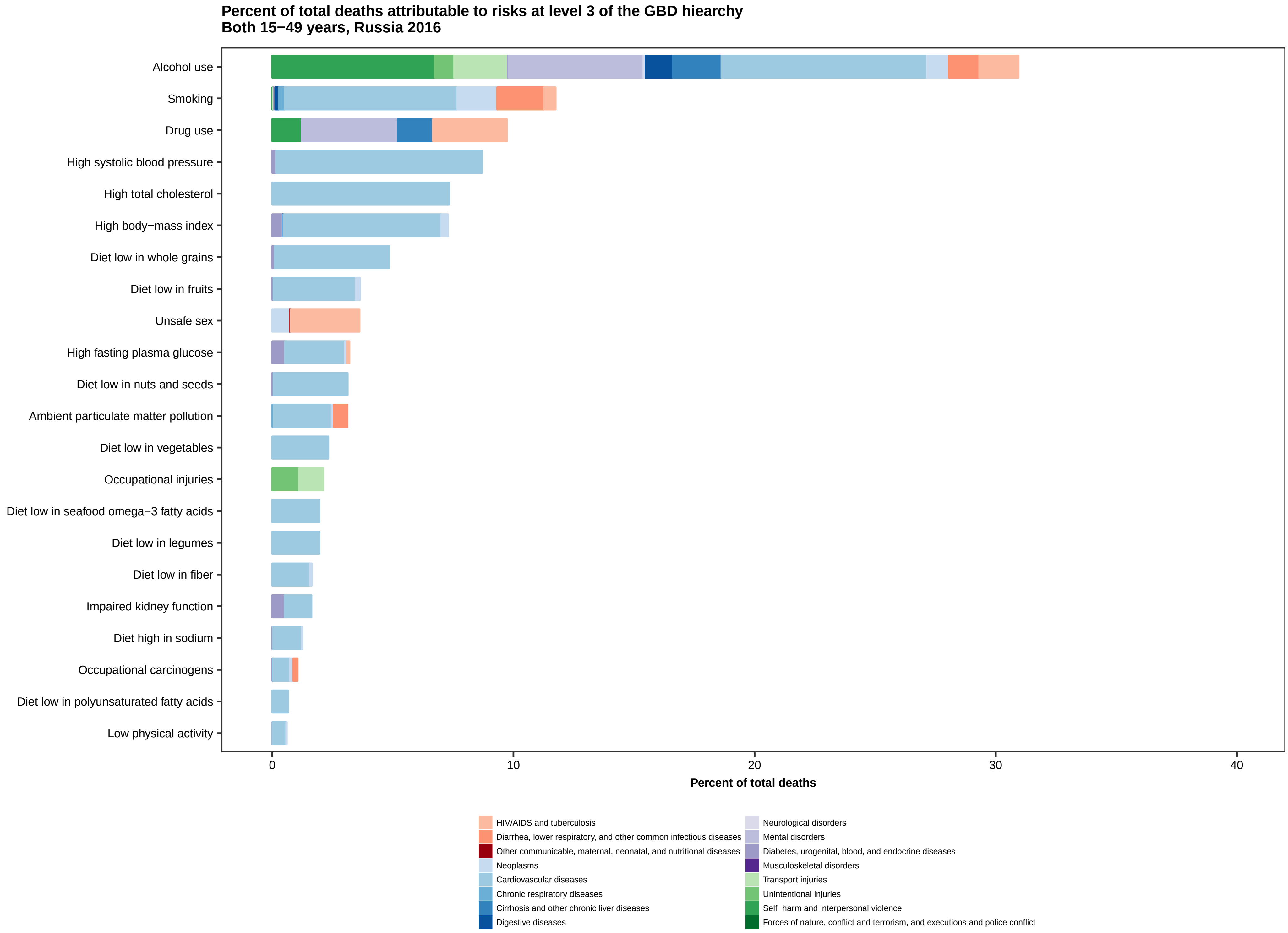

Risks attributable to less than 1% of total deathss omitted

Appendix Table 1. Number and age-standardised rate of deaths and DALYs, and the percent change from 1980 to 2016

	Deaths per 100,000	Number of deaths	DALYS per 100,000	Number of DALYs	percent change
All causes	1,009.4<br>785.9 to 1,311.1	1,997,038<br>1,541,907 to 2,591,653	34,980.8<br>28,197.5 to 43,102.1	62,885,482<br>50,354,314 to 77,786,870	-16.6<br>-33.8 to 9.4
Communicable, maternal, neonatal, and nutritional diseases	47.7<br>39.6 to 58.2	79,797<br>64,623 to 98,966	3,009.4<br>2,585.5 to 3,456.4	4,380,101<br>3,728,380 to 5,100,529	-25.5<br>-37.9 to -8.8
HIV/AIDS and tuberculosis	18.3<br>15.7 to 21.3	30,875<br>26,440 to 36,294	905.4<br>797.0 to 1,036.0	1,478,772<br>1,289,707 to 1,702,175	80.7<br>48.2 to 116.9
Tuberculosis	7.2<br>5.0 to 10.2	13,123<br>9,081 to 18,405	300.4<br>209.9 to 424.5	518,909<br>360,127 to 730,715	-31.1<br>-51.5 to -5.7
Drug-susceptible tuberculosis	4.5<br>3.0 to 6.5	8,079<br>5,386 to 11,778	187.0<br>127.6 to 272.1	322,780<br>219,777 to 469,814	-57.4<br>-70.2 to -38.3
Multidrug-resistant tuberculosis without extensive drug resistance	1.8<br>1.1 to 2.6	3,196<br>1,950 to 4,759	72.1<br>44.1 to 108.0	124,767<br>75,528 to 186,505	5,010.3<br>2,103.4 to 9,933.1
Extensively drug-resistant tuberculosis	1.0<br>0.6 to 1.5	1,848<br>1,131 to 2,743	41.2<br>25.0 to 62.2	71,362<br>43,033 to 106,795	-
HIV/AIDS	11.0<br>10.1 to 12.1	17,752<br>16,233 to 19,578	605.0<br>548.3 to 672.5	959,863<br>869,606 to 1,072,136	-
HIV-AIDS â€“ Drug-susceptible Tuberculosis	0.4<br>0.2 to 0.7	653<br>352 to 1,137	21.4<br>11.8 to 37.0	33,994<br>18,690 to 58,658	-
HIV/AIDS - Multidrug-resistant Tuberculosis without extensive drug resistance	0.2<br>0.1 to 0.3	329<br>174 to 559	10.6<br>5.7 to 18.0	16,858<br>8,961 to 28,507	-
HIV/AIDS - Extensively drug-resistant Tuberculosis	0.1<br>0.1 to 0.2	190<br>99 to 324	6.1<br>3.2 to 10.4	9,692<br>5,070 to 16,478	-
HIV/AIDS resulting in other diseases	10.3<br>9.3 to 11.4	16,580<br>14,896 to 18,431	566.8<br>506.7 to 635.8	899,319<br>802,363 to 1,011,861	-
Diarrhea, lower respiratory, and other common infectious diseases	22.6<br>17.0 to 29.6	41,162<br>30,298 to 54,589	1,163.9<br>917.3 to 1,442.1	1,800,962<br>1,379,857 to 2,280,520	-29.2<br>-45.8 to -6.6
Diarrheal diseases	0.3<br>0.2 to 0.4	439<br>347 to 545	91.2<br>66.7 to 119.7	111,524<br>81,554 to 145,633	-89.7<br>-92.0 to -86.4
Intestinal infectious diseases	0.0<br>0.0 to 0.0	9<br>3 to 21	0.3<br>0.1 to 0.6	386<br>136 to 887	-67.8<br>-77.4 to -53.3
Typhoid fever	0.0<br>0.0 to 0.0	5<br>1 to 11	0.2<br>0.1 to 0.4	221<br>74 to 499	-81.7<br>-86.3 to -76.0
Paratyphoid fever	0.0<br>0.0 to 0.0	4<br>1 to 11	0.1<br>0.0 to 0.3	157<br>44 to 397	120.3<br>55.8 to 231.1
Other intestinal infectious diseases	0.0<br>0.0 to 0.0	0<br>0 to 1	0.0<br>0.0 to 0.0	8<br>3 to 16	-39.4<br>-66.4 to -4.3
Lower respiratory infections	20.4<br>15.1 to 26.9	37,613<br>27,719 to 50,026	742.5<br>557.3 to 974.6	1,238,472<br>903,679 to 1,641,732	-15.9<br>-37.5 to 12.4
Upper respiratory infections	0.0<br>0.0 to 0.0	27<br>13 to 47	172.9<br>104.9 to 268.8	233,662<br>141,869 to 359,796	-20.9<br>-68.3 to 79.5
Otitis media	0.0<br>0.0 to 0.0	3<br>2 to 5	32.6<br>20.1 to 49.0	39,707<br>24,885 to 58,956	-72.1<br>-84.2 to -53.6
Meningitis	1.0<br>0.7 to 1.2	1,534<br>1,160 to 2,019	61.4<br>49.5 to 74.2	85,083<br>67,234 to 104,629	-73.7<br>-80.4 to -65.4
Pneumococcal meningitis	0.1<br>0.0 to 0.1	117<br>85 to 159	7.5<br>5.7 to 9.4	11,194<br>8,525 to 14,110	-51.8<br>-63.3 to -37.4

H influenzae type B meningitis	0.1<br>0.1 to 0.1	114<br>87 to 147	6.1<br>5.0 to 7.4	8,231<br>6,637 to 9,958	-67.8<br>-74.5 to -59.5
Meningococcal meningitis	0.5<br>0.4 to 0.6	731<br>564 to 939	30.5<br>24.8 to 36.9	39,195<br>31,564 to 48,020	-80.8<br>-86.2 to -74.0
Other meningitis	0.3<br>0.2 to 0.4	572<br>407 to 770	17.2<br>13.2 to 21.6	26,464<br>19,923 to 34,008	-53.6<br>-64.6 to -38.8
Encephalitis	0.9<br>0.7 to 1.4	1,485<br>1,052 to 2,248	56.5<br>44.1 to 79.3	83,072<br>64,375 to 116,079	-3.4<br>-25.2 to 24.3
Diphtheria	0.0<br>0.0 to 0.0	5<br>3 to 8	0.2<br>0.1 to 0.3	230<br>144 to 360	-96.7<br>-98.4 to -93.6
Whooping cough	0.0<br>0.0 to 0.0	9<br>4 to 30	1.9<br>1.1 to 3.5	1,938<br>1,133 to 3,625	-33.9<br>-64.8 to 17.3
Tetanus	0.0<br>0.0 to 0.0	4<br>3 to 7	0.1<br>0.1 to 0.1	124<br>85 to 190	-95.8<br>-96.9 to -94.0
Measles	0.0<br>0.0 to 0.0	3<br>2 to 5	0.3<br>0.2 to 0.4	268<br>186 to 376	-92.5<br>-95.6 to -88.7
Varicella and herpes zoster	0.0<br>0.0 to 0.0	29<br>12 to 46	4.3<br>2.5 to 6.0	6,494<br>4,058 to 9,332	-5.1<br>-32.2 to 23.2
Neglected tropical diseases and malaria	0.1<br>0.0 to 0.1	115<br>65 to 154	21.6<br>14.1 to 32.2	37,273<br>23,394 to 58,302	-4.7<br>-26.0 to 29.6
Malaria	0.0<br>0.0 to 0.0	0<br>0 to 0	0.0<br>0.0 to 0.0	0<br>0 to 0	-
Chagas disease	0.0<br>0.0 to 0.0	0<br>0 to 0	0.0<br>0.0 to 0.0	0<br>0 to 0	-
Leishmaniasis	0.0<br>0.0 to 0.0	0<br>0 to 0	0.0<br>0.0 to 0.0	0<br>0 to 0	-
Visceral leishmaniasis	0.0<br>0.0 to 0.0	0<br>0 to 0	0.0<br>0.0 to 0.0	0<br>0 to 0	-
African trypanosomiasis	0.0<br>0.0 to 0.0	0<br>0 to 0	0.0<br>0.0 to 0.0	0<br>0 to 0	-
Schistosomiasis	0.0<br>0.0 to 0.0	0<br>0 to 0	0.0<br>0.0 to 0.0	0<br>0 to 0	-
Cysticercosis	0.0<br>0.0 to 0.0	4<br>3 to 5	0.9<br>0.1 to 2.4	1,685<br>114 to 4,410	-65.9<br>-74.8 to -53.1
Cystic echinococcosis	0.0<br>0.0 to 0.0	35<br>25 to 47	1.5<br>0.7 to 4.0	2,568<br>1,195 to 6,834	-65.0<br>-73.4 to -52.6
Dengue	0.0<br>0.0 to 0.0	0<br>0 to 0	0.0<br>0.0 to 0.0	0<br>0 to 0	-
Yellow fever	0.0<br>0.0 to 0.0	0<br>0 to 0	0.0<br>0.0 to 0.0	0<br>0 to 0	-
Rabies	0.0<br>0.0 to 0.0	3<br>2 to 4	0.1<br>0.1 to 0.1	123<br>86 to 173	7.6<br>-37.9 to 66.7
Intestinal nematode infections	0.0<br>0.0 to 0.0	0<br>0 to 0	0.0<br>0.0 to 0.0	0<br>0 to 0	-
Ascariasis	0.0<br>0.0 to 0.0	0<br>0 to 0	0.0<br>0.0 to 0.0	0<br>0 to 0	-
Ebola	0.0<br>0.0 to 0.0	0<br>0 to 0	0.0<br>0.0 to 0.0	0<br>0 to 0	-

Zika virus	0.0<br>0.0 to 0.0	0<br>0 to 0	0.0<br>0.0 to 0.0	0<br>0 to 0	-
Other neglected tropical diseases	0.0<br>0.0 to 0.1	74<br>26 to 103	2.7<br>1.0 to 3.7	3,556<br>1,206 to 4,962	163.4<br>88.9 to 256.3
Maternal disorders	0.2<br>0.1 to 0.4	337<br>178 to 588	19.7<br>13.2 to 29.6	29,241<br>19,601 to 44,381	-80.7<br>-89.8 to -64.5
Maternal hemorrhage	0.0<br>0.0 to 0.1	63<br>31 to 113	4.4<br>2.9 to 6.5	6,698<br>4,347 to 9,969	-77.0<br>-87.8 to -53.3
Maternal sepsis and other maternal infections	0.0<br>0.0 to 0.0	24<br>12 to 43	1.9<br>1.2 to 3.0	2,762<br>1,687 to 4,332	-70.2<br>-83.4 to -42.9
Maternal hypertensive disorders	0.0<br>0.0 to 0.0	40<br>20 to 71	3.3<br>2.2 to 5.1	4,860<br>3,127 to 7,339	-80.3<br>-90.0 to -64.9
Maternal obstructed labor and uterine rupture	0.0<br>0.0 to 0.0	5<br>2 to 10	0.5<br>0.3 to 0.9	804<br>420 to 1,352	8.9<br>-47.0 to 126.7
Maternal abortion, miscarriage, and ectopic pregnancy	0.0<br>0.0 to 0.1	63<br>33 to 113	3.2<br>1.9 to 5.0	4,725<br>2,870 to 7,604	-89.6<br>-94.5 to -80.5
Indirect maternal deaths	0.0<br>0.0 to 0.1	70<br>36 to 127	2.7<br>1.4 to 4.8	3,965<br>2,061 to 7,221	-84.3<br>-92.5 to -70.8
Late maternal deaths	0.0<br>0.0 to 0.0	3<br>1 to 5	0.1<br>0.1 to 0.2	149<br>72 to 286	-88.6<br>-94.2 to -78.7
Maternal deaths aggravated by HIV/AIDS	0.0<br>0.0 to 0.0	1<br>0 to 1	0.0<br>0.0 to 0.0	34<br>12 to 76	-
Other maternal disorders	0.0<br>0.0 to 0.1	68<br>35 to 121	3.5<br>2.2 to 5.4	5,243<br>3,290 to 8,133	-29.0<br>-61.4 to 33.8
Neonatal disorders	5.5<br>4.7 to 6.2	5,599<br>4,792 to 6,318	681.9<br>582.2 to 790.3	750,777<br>636,852 to 884,474	-70.6<br>-74.6 to -65.2
Neonatal preterm birth complications	2.0<br>1.6 to 2.4	2,060<br>1,654 to 2,461	238.9<br>197.6 to 281.1	260,574<br>215,017 to 308,983	-75.4<br>-80.3 to -68.8
Neonatal encephalopathy due to birth asphyxia and trauma	1.4<br>1.1 to 1.7	1,452<br>1,137 to 1,765	203.8<br>155.8 to 260.7	231,521<br>172,861 to 305,751	-78.4<br>-82.8 to -72.5
Neonatal sepsis and other neonatal infections	0.9<br>0.6 to 1.4	890<br>653 to 1,414	120.9<br>83.8 to 184.7	135,380<br>91,195 to 217,576	-3.2<br>-34.0 to 30.7
Hemolytic disease and other neonatal jaundice	0.1<br>0.0 to 0.1	63<br>42 to 95	14.8<br>11.3 to 19.2	18,248<br>14,013 to 23,648	-84.8<br>-91.8 to -74.4
Other neonatal disorders	1.1<br>0.9 to 1.4	1,134<br>886 to 1,408	103.5<br>81.6 to 126.8	105,053<br>82,779 to 128,522	-55.3<br>-69.7 to -39.5
Nutritional deficiencies	0.4<br>0.3 to 0.5	751<br>564 to 991	130.3<br>84.4 to 193.2	166,755<br>111,166 to 248,655	-48.7<br>-60.0 to -27.8
Protein-energy malnutrition	0.1<br>0.1 to 0.2	213<br>122 to 372	19.4<br>12.7 to 28.1	22,612<br>15,341 to 32,197	-68.2<br>-75.9 to -53.5
Iodine deficiency	0.0<br>0.0 to 0.0	27<br>2 to 77	15.7<br>9.6 to 23.3	26,257<br>16,315 to 38,334	-54.3<br>-97.1 to 44.8
Iron-deficiency anemia	0.0<br>0.0 to 0.0	32<br>19 to 75	88.1<br>51.6 to 140.6	105,926<br>62,808 to 171,859	-89.0<br>-92.0 to -83.9
Other nutritional deficiencies	0.2<br>0.2 to 0.3	479<br>314 to 652	7.0<br>4.7 to 9.5	11,960<br>7,811 to 16,187	9.6<br>-20.9 to 61.7
Other communicable, maternal, neonatal, and nutritional diseases	0.7<br>0.5 to 0.9	959<br>720 to 1,212	85.9<br>63.2 to 115.4	115,275<br>85,937 to 157,258	-29.1<br>-45.1 to -9.1

Sexually transmitted diseases excluding HIV	0.1<br>0.1 to 0.2	210<br>130 to 323	40.9<br>26.9 to 62.4	61,939<br>41,079 to 93,684	-58.5<br>-77.4 to -33.7
Syphilis	0.1<br>0.0 to 0.1	89<br>52 to 145	9.2<br>5.8 to 13.9	13,863<br>9,701 to 19,124	-71.7<br>-86.4 to -54.0
Chlamydial infection	0.0<br>0.0 to 0.0	23<br>12 to 38	6.4<br>3.8 to 10.6	9,891<br>6,010 to 16,250	-9.4<br>-43.7 to 52.1
Gonococcal infection	0.0<br>0.0 to 0.1	66<br>36 to 109	8.8<br>5.2 to 14.3	12,040<br>7,320 to 19,306	-25.0<br>-53.3 to 23.0
Other sexually transmitted diseases	0.0<br>0.0 to 0.0	32<br>17 to 53	11.5<br>7.4 to 17.9	17,413<br>11,336 to 26,647	-24.6<br>-53.5 to 25.4
Acute hepatitis	0.1<br>0.1 to 0.2	246<br>182 to 329	7.5<br>5.8 to 9.5	10,852<br>8,280 to 14,198	-69.4<br>-76.4 to -56.5
Acute hepatitis A	0.0<br>0.0 to 0.0	20<br>15 to 27	1.7<br>1.2 to 2.2	1,830<br>1,364 to 2,377	-85.9<br>-90.7 to -79.2
Acute hepatitis B	0.1<br>0.1 to 0.2	201<br>142 to 274	5.0<br>3.6 to 6.7	7,975<br>5,712 to 10,836	-60.8<br>-71.2 to -43.1
Acute hepatitis C	0.0<br>0.0 to 0.0	8<br>5 to 12	0.2<br>0.1 to 0.3	316<br>216 to 443	-57.5<br>-68.7 to -38.5
Acute hepatitis E	0.0<br>0.0 to 0.0	17<br>12 to 24	0.6<br>0.4 to 0.8	730<br>531 to 987	-78.9<br>-84.7 to -70.9
Other unspecified infectious diseases	0.4<br>0.3 to 0.5	502<br>333 to 638	37.5<br>23.5 to 48.9	42,484<br>27,857 to 55,007	115.9<br>68.1 to 171.7
Non-communicable diseases	858.7<br>663.0 to 1,107.3	1,742,519<br>1,339,352 to 2,250,680	26,020.5<br>20,835.1 to 31,997.4	48,938,521<br>39,218,978 to 60,458,684	-14.5<br>-32.5 to 13.2
Neoplasms	147.5<br>111.6 to 195.4	305,543<br>231,764 to 403,970	3,585.1<br>2,703.2 to 4,798.4	7,151,792<br>5,383,879 to 9,555,005	-16.8<br>-35.1 to 11.0
Lip and oral cavity cancer	2.9<br>2.1 to 3.9	6,005<br>4,270 to 8,046	76.6<br>53.8 to 104.0	155,928<br>109,164 to 209,940	9.1<br>-16.8 to 44.7
Nasopharynx cancer	0.3<br>0.2 to 0.4	643<br>461 to 863	9.7<br>6.9 to 13.1	18,501<br>13,086 to 24,956	28.8<br>-6.2 to 70.5
Other pharynx cancer	1.8<br>1.2 to 2.4	3,691<br>2,542 to 5,049	49.3<br>33.8 to 67.9	101,830<br>69,675 to 139,804	31.2<br>-6.0 to 77.1
Esophageal cancer	3.7<br>2.7 to 4.9	7,916<br>5,673 to 10,463	88.5<br>61.9 to 119.3	185,337<br>129,088 to 249,422	-32.1<br>-50.0 to -9.2
Stomach cancer	17.0<br>12.8 to 22.3	35,534<br>26,847 to 46,381	376.5<br>278.8 to 505.6	770,711<br>575,054 to 1,030,220	-60.4<br>-69.5 to -47.5
Colon and rectum cancer	19.6<br>14.8 to 25.7	41,391<br>30,944 to 54,140	402.7<br>297.7 to 531.8	833,634<br>617,041 to 1,101,547	17.5<br>-7.8 to 63.6
Liver cancer	5.1<br>3.8 to 6.7	10,640<br>7,995 to 13,985	116.4<br>85.2 to 155.5	236,911<br>175,402 to 315,908	15.5<br>-10.3 to 52.9
Liver cancer due to hepatitis B	1.8<br>1.3 to 2.5	3,836<br>2,692 to 5,234	45.0<br>30.9 to 62.2	90,554<br>62,380 to 125,732	5.4<br>-18.7 to 39.6
Liver cancer due to hepatitis C	0.6<br>0.5 to 0.8	1,316<br>995 to 1,767	12.2<br>9.0 to 16.3	25,625<br>18,837 to 34,586	14.6<br>-12.8 to 55.0
Liver cancer due to alcohol use	1.8<br>1.3 to 2.4	3,695<br>2,705 to 4,972	38.7<br>28.0 to 52.9	79,858<br>57,708 to 108,556	45.3<br>10.5 to 99.0
Liver cancer due to other causes	0.9<br>0.6 to 1.1	1,793<br>1,307 to 2,362	20.5<br>14.8 to 27.3	40,874<br>29,275 to 54,909	-4.5<br>-25.8 to 28.2

Gallbladder and biliary tract cancer	1.7	3,546	33.3	69,222	-54.9
	1.2 to 2.3	2,609 to 4,812	24.4 to 45.6	50,881 to 94,895	-66.1 to -35.2
Pancreatic cancer	8.4	17,645	187.8	385,950	30.2
	6.3 to 11.3	13,259 to 23,575	139.4 to 253.5	287,502 to 520,887	-1.7 to 71.5
Larynx cancer	2.4	4,959	61.1	127,361	-28.6
	1.6 to 3.3	3,439 to 6,825	41.7 to 84.9	87,068 to 177,078	-50.8 to -0.8
Tracheal, bronchus, and lung cancer	24.7	52,152	581.2	1,216,078	-28.4
	17.7 to 32.2	37,268 to 67,758	409.2 to 780.5	856,245 to 1,631,400	-47.2 to -4.9
Malignant skin melanoma	2.2	4,390	61.0	116,733	30.4
	1.5 to 3.0	3,004 to 6,044	42.0 to 83.9	80,735 to 159,565	-1.8 to 90.9
Non-melanoma skin cancer	1.1	2,225	20.1	40,737	20.8
	0.8 to 1.4	1,717 to 2,868	15.1 to 26.9	30,526 to 54,297	-3.8 to 54.5
Non-melanoma skin cancer (squamous-cell carcinoma)	1.1	2,225	20.1	40,709	20.8
	0.8 to 1.4	1,717 to 2,868	15.0 to 26.9	30,518 to 54,262	-3.8 to 54.5
Breast cancer	11.6	23,791	297.8	595,322	18.3
	7.3 to 17.8	15,140 to 36,677	190.5 to 465.4	381,822 to 926,220	-24.2 to 89.1
Cervical cancer	3.5	6,916	111.7	207,109	-46.9
	2.1 to 5.6	4,210 to 10,891	66.4 to 182.2	123,606 to 333,103	-66.7 to -14.9
Uterine cancer	3.2	6,658	77.5	159,062	68.4
	2.0 to 4.9	4,197 to 10,170	49.3 to 115.4	102,073 to 238,066	0.7 to 171.5
Ovarian cancer	4.3	8,822	112.6	223,712	-16.4
	2.6 to 6.6	5,319 to 13,615	68.0 to 175.0	135,432 to 345,816	-50.6 to 34.8
Prostate cancer	5.6	11,836	104.1	220,902	157.7
	3.8 to 7.5	8,062 to 16,085	71.5 to 140.8	151,847 to 298,986	89.1 to 246.9
Testicular cancer	0.2	340	8.9	14,530	-35.9
	0.1 to 0.3	217 to 480	5.6 to 12.9	9,088 to 20,985	-58.2 to -2.7
Kidney cancer	4.6	9,617	112.8	228,686	75.4
	3.4 to 6.0	7,125 to 12,565	83.3 to 148.5	168,656 to 301,242	38.3 to 134.6
Bladder cancer	3.6	7,563	69.3	145,353	-7.1
	2.7 to 4.6	5,714 to 9,829	51.8 to 91.6	108,729 to 192,113	-28.8 to 19.2
Brain and nervous system cancer	4.0	7,469	144.3	243,185	-7.8
	2.9 to 5.4	5,421 to 10,134	108.2 to 192.0	179,921 to 325,252	-31.5 to 27.0
Thyroid cancer	0.6	1,263	14.5	29,243	-11.6
	0.4 to 0.8	900 to 1,733	10.8 to 19.4	21,677 to 39,338	-34.2 to 26.7
Mesothelioma	0.3	691	8.6	16,950	-9.7
	0.2 to 0.4	507 to 922	6.1 to 11.6	12,191 to 22,916	-32.1 to 28.6
Hodgkin lymphoma	0.6	1,066	24.5	40,357	-52.2
	0.4 to 0.8	767 to 1,466	17.8 to 33.6	29,094 to 55,308	-64.4 to -32.7
Non-Hodgkin lymphoma	2.4	4,752	71.9	129,565	1.2
	1.8 to 3.3	3,480 to 6,410	52.5 to 95.5	94,728 to 172,747	-22.8 to 33.3
Multiple myeloma	1.3	2,662	31.0	63,391	17.6
	0.9 to 1.8	1,916 to 3,652	22.1 to 42.7	45,414 to 86,642	-16.0 to 70.1
Leukemia	4.4	8,324	139.9	233,844	-19.9
	3.3 to 5.7	6,250 to 10,936	108.6 to 182.5	178,222 to 305,292	-38.1 to 4.7
Acute lymphoid leukemia	0.8	1,350	38.9	52,487	-36.5
	0.6 to 1.1	1,009 to 1,770	30.9 to 48.8	40,655 to 67,725	-49.8 to -15.1
Chronic lymphoid leukemia	0.9	1,959	19.9	41,555	24.6
	0.7 to 1.2	1,446 to 2,619	14.7 to 26.7	30,610 to 55,777	-6.3 to 62.1

Acute myeloid leukemia	1.3<br>1.0 to 1.8	2,480<br>1,820 to 3,350	45.3<br>33.1 to 60.5	75,630<br>55,067 to 101,405	5.8<br>-20.3 to 40.4
Chronic myeloid leukemia	0.3<br>0.2 to 0.5	691<br>501 to 937	9.1<br>6.5 to 12.5	17,331<br>12,412 to 23,869	-41.9<br>-59.5 to -21.2
Other leukemia	0.9<br>0.7 to 1.2	1,843<br>1,359 to 2,429	26.7<br>20.2 to 34.9	46,841<br>34,797 to 61,845	-38.8<br>-53.4 to -15.4
Other neoplasms	6.6<br>4.9 to 8.9	13,036<br>9,758 to 17,584	191.5<br>142.8 to 254.6	341,646<br>252,011 to 459,118	7.4<br>-16.6 to 46.9
Cardiovascular diseases	562.8<br>438.3 to 721.3	1,150,431<br>892,130 to 1,479,670	9,998.2<br>7,675.5 to 12,985.0	20,348,866<br>15,677,148 to 26,401,671	-13.6<br>-31.4 to 14.8
Rheumatic heart disease	2.1<br>1.5 to 2.9	4,301<br>3,076 to 5,889	55.7<br>39.8 to 76.1	109,043<br>77,819 to 149,391	-72.8<br>-80.0 to -62.4
Ischemic heart disease	327.1<br>255.1 to 416.6	670,801<br>521,602 to 857,942	5,240.0<br>3,985.9 to 6,825.7	10,823,042<br>8,238,153 to 14,113,901	-13.7<br>-30.7 to 13.2
Stroke	168.0<br>131.5 to 215.3	345,861<br>267,315 to 444,861	2,939.5<br>2,300.8 to 3,732.5	6,082,727<br>4,773,920 to 7,736,480	-25.1<br>-41.5 to 0.7
Ischemic stroke	124.9<br>97.0 to 159.3	258,005<br>199,821 to 330,603	2,009.7<br>1,588.8 to 2,514.7	4,228,949<br>3,338,120 to 5,297,810	-26.8<br>-42.5 to -1.5
Intracerebral hemorrhage	43.1<br>32.4 to 56.5	87,856<br>66,274 to 115,435	929.8<br>695.8 to 1,233.7	1,853,779<br>1,393,393 to 2,447,712	-19.7<br>-39.8 to 10.0
Hypertensive heart disease	11.1<br>7.5 to 18.6	22,894<br>15,465 to 38,505	197.5<br>137.7 to 317.0	408,998<br>284,009 to 656,535	100.9<br>51.7 to 181.6
Cardiomyopathy and myocarditis	34.0<br>23.0 to 46.1	64,720<br>44,327 to 88,101	1,100.3<br>762.2 to 1,520.1	1,998,050<br>1,397,323 to 2,743,864	46.1<br>9.9 to 99.1
Myocarditis	0.6<br>0.3 to 0.9	1,138<br>514 to 1,700	16.7<br>11.7 to 24.5	26,848<br>18,080 to 38,354	7.8<br>-23.4 to 53.8
Alcoholic cardiomyopathy	24.9<br>17.3 to 34.4	47,550<br>33,110 to 65,686	855.8<br>581.3 to 1,199.2	1,567,584<br>1,071,435 to 2,200,284	21.8<br>-9.4 to 66.7
Other cardiomyopathy	8.4<br>3.8 to 12.1	16,032<br>7,358 to 23,173	227.8<br>122.9 to 331.5	403,618<br>218,225 to 585,699	231.1<br>146.1 to 348.5
Atrial fibrillation and flutter	4.0<br>2.8 to 5.6	8,153<br>5,608 to 11,461	92.1<br>69.5 to 119.3	193,043<br>145,142 to 249,660	11.9<br>-16.3 to 51.0
Aortic aneurysm	3.7<br>2.8 to 4.8	7,684<br>5,793 to 9,970	78.1<br>57.6 to 104.1	158,078<br>116,488 to 209,248	285.9<br>203.1 to 404.1
Peripheral artery disease	4.6<br>3.1 to 7.8	9,499<br>6,388 to 15,825	70.7<br>49.6 to 104.8	149,180<br>105,118 to 220,905	36.3<br>6.9 to 80.5
Endocarditis	1.2<br>0.8 to 1.9	2,211<br>1,479 to 3,622	39.0<br>26.4 to 60.2	69,111<br>46,406 to 108,352	-37.7<br>-53.6 to -10.1
Other cardiovascular and circulatory diseases	7.1<br>5.4 to 9.2	14,307<br>10,800 to 18,825	185.3<br>139.8 to 243.5	357,593<br>269,950 to 470,970	285.3<br>188.0 to 435.8
Chronic respiratory diseases	18.0<br>13.7 to 22.8	37,624<br>28,675 to 47,654	586.7<br>467.9 to 725.4	1,091,075<br>873,206 to 1,349,157	-69.6<br>-76.3 to -59.5
Chronic obstructive pulmonary disease	16.0<br>12.3 to 20.2	33,573<br>25,645 to 42,469	341.4<br>265.5 to 428.5	703,194<br>545,570 to 883,291	-66.3<br>-74.1 to -54.5
Pneumoconiosis	0.1<br>0.1 to 0.1	221<br>164 to 288	3.1<br>2.4 to 3.9	6,445<br>4,959 to 8,088	-59.1<br>-70.3 to -45.4
Silicosis	0.0<br>0.0 to 0.0	28<br>17 to 44	0.4<br>0.3 to 0.6	840<br>606 to 1,174	-71.6<br>-80.8 to -61.2

Asbestosis	0.0<br>0.0 to 0.0	29<br>15 to 43	0.7<br>0.5 to 0.9	1,332<br>962 to 1,764	-68.7<br>-77.7 to -57.9
Coal workers pneumoconiosis	0.0<br>0.0 to 0.0	28<br>6 to 44	0.4<br>0.2 to 0.5	755<br>346 to 1,083	148.5<br>55.9 to 254.0
Other pneumoconiosis	0.1<br>0.0 to 0.1	136<br>97 to 188	1.7<br>1.3 to 2.1	3,518<br>2,637 to 4,456	-60.0<br>-72.7 to -45.3
Asthma	1.2<br>0.8 to 1.9	2,377<br>1,685 to 3,817	209.8<br>144.9 to 291.1	324,927<br>228,163 to 447,734	-87.8<br>-91.2 to -82.3
Interstitial lung disease and pulmonary sarcoidosis	0.4<br>0.2 to 0.5	773<br>475 to 1,079	18.2<br>12.8 to 24.0	33,215<br>23,100 to 43,613	9.2<br>-17.0 to 57.8
Other chronic respiratory diseases	0.3<br>0.2 to 0.6	680<br>441 to 1,194	14.2<br>10.3 to 22.2	23,294<br>16,521 to 37,157	-77.9<br>-83.7 to -70.5
Cirrhosis and other chronic liver diseases	25.9<br>18.7 to 35.1	49,991<br>36,353 to 67,356	916.4<br>661.1 to 1,250.7	1,682,255<br>1,217,503 to 2,285,151	126.9<br>67.4 to 196.6
Cirrhosis and other chronic liver diseases due to hepatitis B	5.1<br>3.5 to 7.0	9,706<br>6,826 to 13,330	180.7<br>123.6 to 253.8	331,010<br>228,902 to 460,502	119.8<br>61.5 to 189.8
Cirrhosis and other chronic liver diseases due to hepatitis C	6.3<br>4.5 to 8.7	12,185<br>8,666 to 16,668	225.1<br>156.1 to 311.8	415,086<br>289,618 to 570,824	131.9<br>71.2 to 206.2
Cirrhosis and other chronic liver diseases due to alcohol use	9.2<br>6.6 to 12.6	17,852<br>12,805 to 24,322	310.4<br>221.4 to 427.6	579,685<br>415,125 to 795,671	133.3<br>73.4 to 202.3
Cirrhosis and other chronic liver diseases due to other causes	5.4<br>3.8 to 7.5	10,247<br>7,166 to 14,430	200.2<br>142.2 to 279.5	356,474<br>252,391 to 498,760	118.1<br>59.5 to 211.5
Digestive diseases	20.2<br>14.5 to 26.9	40,466<br>29,239 to 53,721	663.8<br>494.6 to 866.3	1,228,472<br>920,190 to 1,606,379	22.3<br>-6.1 to 62.2
Peptic ulcer disease	4.0<br>2.8 to 5.3	8,130<br>5,612 to 10,856	127.8<br>92.6 to 166.8	245,450<br>177,480 to 320,483	5.1<br>-19.6 to 37.7
Gastritis and duodenitis	0.2<br>0.1 to 0.3	439<br>294 to 669	36.4<br>25.0 to 51.8	59,655<br>41,689 to 84,667	26.6<br>-7.9 to 73.1
Appendicitis	0.3<br>0.2 to 0.4	514<br>358 to 852	11.3<br>8.3 to 17.1	17,852<br>13,104 to 27,752	-75.2<br>-81.1 to -67.4
Paralytic ileus and intestinal obstruction	1.6<br>1.1 to 2.1	3,060<br>2,154 to 4,203	39.5<br>28.1 to 54.9	70,665<br>49,514 to 98,221	-9.7<br>-29.1 to 18.7
Inguinal, femoral, and abdominal hernia	0.6<br>0.4 to 1.0	1,221<br>802 to 2,162	57.0<br>40.7 to 75.0	101,883<br>74,059 to 133,071	-56.6<br>-66.2 to -39.6
Inflammatory bowel disease	0.6<br>0.3 to 0.8	1,121<br>650 to 1,562	29.9<br>22.5 to 38.2	55,679<br>41,925 to 71,195	9.6<br>-17.1 to 52.0
Vascular intestinal disorders	4.5<br>3.3 to 5.9	9,422<br>6,928 to 12,464	76.4<br>55.6 to 104.7	158,680<br>115,682 to 215,803	70.7<br>30.2 to 126.0
Gallbladder and biliary diseases	1.5<br>1.1 to 2.1	3,201<br>2,274 to 4,363	32.8<br>24.9 to 43.1	66,544<br>50,425 to 87,453	-32.0<br>-47.1 to -9.1
Pancreatitis	5.9<br>3.9 to 8.3	11,129<br>7,330 to 15,646	209.7<br>135.8 to 298.3	374,528<br>243,034 to 532,074	105.2<br>51.2 to 174.1
Other digestive diseases	1.1<br>0.7 to 1.6	2,228<br>1,311 to 3,172	42.9<br>28.7 to 59.1	77,538<br>51,524 to 106,446	110.8<br>57.6 to 191.3
Neurological disorders	34.0<br>25.1 to 44.9	67,556<br>49,355 to 90,385	1,380.5<br>1,028.0 to 1,757.3	2,393,339<br>1,791,253 to 3,024,991	-0.8<br>-22.0 to 28.8
Alzheimer disease and other dementias	28.0<br>20.5 to 37.4	55,562<br>39,767 to 75,905	325.1<br>243.2 to 429.3	685,106<br>508,315 to 920,093	1.9<br>-19.9 to 31.2

Parkinson disease	4.0<br>2.8 to 5.6	8,516<br>5,836 to 12,064	61.8<br>43.1 to 85.4	131,691<br>91,670 to 180,532	9.5<br>-14.0 to 44.3
Epilepsy	0.6<br>0.4 to 0.7	882<br>636 to 1,209	83.2<br>35.3 to 162.5	120,960<br>51,274 to 236,133	-69.3<br>-76.7 to -59.5
Multiple sclerosis	0.4<br>0.3 to 0.7	754<br>480 to 1,259	29.2<br>21.6 to 40.6	51,657<br>38,194 to 71,187	-10.3<br>-37.1 to 32.8
Motor neuron disease	0.2<br>0.2 to 0.3	486<br>357 to 655	8.3<br>6.3 to 11.0	15,218<br>11,485 to 20,363	20.0<br>-10.4 to 63.8
Other neurological disorders	0.8<br>0.6 to 1.1	1,356<br>1,011 to 1,790	51.4<br>38.1 to 69.8	72,296<br>53,819 to 96,703	18.5<br>-4.6 to 59.3
Mental and substance use disorders	27.6<br>19.2 to 38.4	49,717<br>34,643 to 69,025	3,429.5<br>2,658.3 to 4,264.2	5,609,614<br>4,372,293 to 6,939,344	-8.5<br>-34.6 to 26.9
Alcohol use disorders	17.3<br>11.8 to 24.2	31,849<br>21,704 to 44,511	1,119.2<br>831.6 to 1,444.9	1,930,474<br>1,439,473 to 2,497,401	8.2<br>-23.1 to 54.8
Drug use disorders	10.3<br>7.0 to 14.1	17,860<br>12,338 to 24,473	750.8<br>562.1 to 957.7	1,213,222<br>910,380 to 1,543,198	-28.3<br>-48.1 to -1.9
Opioid use disorders	7.0<br>4.7 to 10.1	12,014<br>8,171 to 17,340	541.9<br>406.6 to 700.8	883,082<br>662,505 to 1,138,336	-41.4<br>-57.3 to -19.1
Cocaine use disorders	0.6<br>0.2 to 0.9	1,092<br>375 to 1,626	42.8<br>23.8 to 60.0	68,965<br>37,287 to 96,464	112.7<br>24.5 to 224.5
Amphetamine use disorders	0.3<br>0.1 to 0.5	586<br>191 to 942	32.4<br>20.4 to 46.2	48,266<br>29,701 to 68,696	397.3<br>216.5 to 687.9
Other drug use disorders	2.3<br>1.1 to 3.4	4,168<br>1,920 to 6,063	125.2<br>71.0 to 172.6	202,063<br>113,597 to 278,086	11.9<br>-21.9 to 59.8
Eating disorders	0.0<br>0.0 to 0.0	7<br>4 to 12	27.4<br>17.3 to 39.4	36,401<br>22,172 to 52,923	136.8<br>31.4 to 322.2
Anorexia nervosa	0.0<br>0.0 to 0.0	6<br>3 to 10	6.6<br>3.9 to 10.1	7,759<br>4,679 to 11,941	191.0<br>49.3 to 413.1
Bulimia nervosa	0.0<br>0.0 to 0.0	1<br>1 to 1	20.8<br>12.3 to 31.5	28,642<br>16,735 to 43,874	3.4<br>-43.4 to 65.6
Diabetes, urogenital, blood, and endocrine diseases	16.0<br>12.2 to 21.2	32,307<br>24,364 to 43,003	1,102.9<br>862.7 to 1,391.6	2,043,512<br>1,597,143 to 2,552,904	21.5<br>-6.2 to 68.8
Diabetes mellitus	5.6<br>4.1 to 7.7	11,608<br>8,457 to 15,916	390.3<br>299.2 to 506.7	778,090<br>594,419 to 1,007,933	42.9<br>5.1 to 106.6
Acute glomerulonephritis	0.1<br>0.1 to 0.1	154<br>113 to 212	3.2<br>2.4 to 4.4	5,438<br>3,946 to 7,456	-84.3<br>-89.5 to -77.8
Chronic kidney disease	5.2<br>4.0 to 6.8	10,369<br>7,834 to 13,595	228.3<br>184.3 to 279.7	437,645<br>353,049 to 536,102	17.9<br>-7.4 to 58.7
Chronic kidney disease due to diabetes mellitus	1.3<br>0.9 to 1.7	2,557<br>1,918 to 3,381	52.2<br>41.3 to 64.3	104,046<br>82,728 to 128,062	44.1<br>10.6 to 101.8
Chronic kidney disease due to hypertension	0.4<br>0.3 to 0.5	699<br>528 to 933	11.4<br>8.9 to 14.3	23,120<br>18,047 to 29,108	45.0<br>13.0 to 93.9
Chronic kidney disease due to glomerulonephritis	2.0<br>1.5 to 2.7	3,920<br>2,898 to 5,238	95.8<br>77.0 to 117.7	177,759<br>143,158 to 218,513	-7.1<br>-28.4 to 27.4
Chronic kidney disease due to other causes	1.6<br>1.2 to 2.1	3,193<br>2,412 to 4,184	69.0<br>56.0 to 84.2	132,721<br>107,550 to 162,810	39.7<br>7.7 to 88.2
Urinary diseases and male infertility	4.0<br>3.0 to 5.2	8,230<br>6,145 to 10,699	161.2<br>124.1 to 204.1	317,433<br>243,430 to 402,485	10.4<br>-15.8 to 50.0

Urinary tract infections	3.2<br>2.3 to 4.2	6,487<br>4,826 to 8,610	75.4<br>56.2 to 100.1	143,900<br>106,436 to 190,628	10.4<br>-15.8 to 51.9
Urolithiasis	0.7<br>0.4 to 0.9	1,376<br>879 to 1,961	18.4<br>13.2 to 24.3	36,633<br>26,024 to 48,669	-4.7<br>-28.8 to 36.5
Other urinary diseases	0.2<br>0.1 to 0.3	367<br>153 to 557	8.1<br>5.2 to 11.0	14,336<br>8,949 to 19,581	134.5<br>57.7 to 241.6
Gynecological diseases	0.1<br>0.0 to 0.1	96<br>28 to 167	170.8<br>117.6 to 242.3	269,082<br>184,721 to 383,211	431.2<br>212.4 to 776.6
Uterine fibroids	0.0<br>0.0 to 0.1	55<br>15 to 98	12.3<br>7.0 to 21.3	21,651<br>12,141 to 37,651	611.1<br>290.7 to 1,318.8
Polycystic ovarian syndrome	0.0<br>0.0 to 0.0	1<br>0 to 2	0.9<br>0.4 to 1.7	1,392<br>627 to 2,612	242.5<br>11.8 to 750.1
Endometriosis	0.0<br>0.0 to 0.0	4<br>1 to 9	7.6<br>4.9 to 11.2	10,945<br>6,990 to 16,294	776.8<br>113.4 to 2,069.6
Genital prolapse	0.0<br>0.0 to 0.0	7<br>2 to 12	10.1<br>5.0 to 18.4	20,286<br>9,997 to 36,997	23.7<br>-33.4 to 116.2
Other gynecological diseases	0.0<br>0.0 to 0.0	29<br>9 to 51	86.6<br>57.2 to 125.3	136,643<br>89,920 to 196,333	639.8<br>308.4 to 1,134.6
Hemoglobinopathies and hemolytic anemias	0.3<br>0.2 to 0.4	568<br>371 to 784	45.6<br>31.4 to 64.3	67,534<br>46,956 to 94,758	65.1<br>30.6 to 127.2
Thalassemias	0.0<br>0.0 to 0.0	20<br>17 to 24	1.7<br>1.4 to 2.0	1,680<br>1,384 to 1,955	173.3<br>118.1 to 257.2
Sickle cell disorders	0.0<br>0.0 to 0.1	59<br>35 to 86	2.5<br>1.6 to 3.5	3,255<br>2,007 to 4,656	380.0<br>256.6 to 586.9
G6PD deficiency	0.0<br>0.0 to 0.1	74<br>42 to 105	2.1<br>1.3 to 2.8	3,235<br>1,990 to 4,413	372.3<br>253.5 to 536.4
Other hemoglobinopathies and hemolytic anemias	0.2<br>0.1 to 0.3	414<br>271 to 583	9.1<br>6.4 to 12.4	15,051<br>10,719 to 20,202	25.6<br>-4.3 to 78.1
Endocrine, metabolic, blood, and immune disorders	0.8<br>0.6 to 1.0	1,282<br>930 to 1,781	103.4<br>77.6 to 133.6	168,291<br>125,059 to 220,072	51.8<br>18.1 to 109.9
Musculoskeletal disorders	0.9<br>0.6 to 1.3	1,712<br>1,154 to 2,473	1,841.5<br>1,340.1 to 2,387.9	3,436,410<br>2,505,473 to 4,448,011	-12.5<br>-40.0 to 34.8
Rheumatoid arthritis	0.3<br>0.2 to 0.6	704<br>439 to 1,179	77.6<br>55.4 to 101.1	149,755<br>107,111 to 195,311	-4.7<br>-39.2 to 51.6
Other musculoskeletal disorders	0.5<br>0.3 to 0.8	1,007<br>592 to 1,493	96.1<br>66.6 to 139.1	166,406<br>116,464 to 238,168	-16.3<br>-40.5 to 26.6
Other non-communicable diseases	5.9<br>4.7 to 7.0	7,172<br>5,370 to 8,885	2,515.9<br>1,869.6 to 3,376.9	3,953,186<br>2,874,026 to 5,391,549	-44.7<br>-53.0 to -34.5
Congenital birth defects	4.5<br>3.8 to 5.8	4,930<br>4,174 to 6,440	521.3<br>447.5 to 631.9	597,951<br>512,868 to 718,121	-56.5<br>-62.6 to -49.5
Neural tube defects	0.1<br>0.1 to 0.1	80<br>62 to 107	24.0<br>18.5 to 30.5	32,167<br>24,453 to 41,397	-92.3<br>-94.3 to -89.4
Congenital heart anomalies	2.2<br>1.8 to 3.2	2,462<br>1,989 to 3,581	181.9<br>147.8 to 259.5	195,637<br>159,996 to 278,112	-67.9<br>-73.3 to -61.0
Orofacial clefts	0.0<br>0.0 to 0.0	6<br>4 to 13	0.9<br>0.6 to 1.4	1,044<br>728 to 1,570	-82.0<br>-92.9 to -56.4
Down syndrome	0.2<br>0.1 to 0.2	191<br>135 to 248	20.6<br>15.3 to 25.9	25,488<br>19,523 to 32,039	118.1<br>70.0 to 166.3

Other chromosomal abnormalities	0.2<br>0.1 to 0.3	218<br>124 to 293	34.5<br>26.3 to 43.6	34,466<br>25,673 to 43,444	398.0<br>216.0 to 584.3
Congenital musculoskeletal and limb anomalies	0.1<br>0.1 to 0.2	80<br>54 to 201	57.8<br>40.7 to 78.1	82,961<br>58,426 to 112,211	-51.9<br>-63.8 to -38.0
Urogenital congenital anomalies	0.1<br>0.0 to 0.1	81<br>54 to 142	10.4<br>7.4 to 15.2	11,184<br>8,009 to 16,322	-23.8<br>-43.3 to -1.3
Digestive congenital anomalies	0.4<br>0.3 to 0.7	442<br>310 to 705	42.8<br>31.8 to 64.0	47,047<br>35,263 to 68,921	-50.0<br>-65.1 to -34.2
Other congenital birth defects	1.3<br>0.9 to 1.6	1,369<br>989 to 1,648	146.4<br>114.8 to 175.8	165,533<br>129,935 to 201,382	-10.7<br>-22.3 to 7.5
Skin and subcutaneous diseases	0.9<br>0.2 to 1.6	1,828<br>361 to 3,164	737.5<br>505.4 to 1,059.6	1,000,465<br>683,909 to 1,435,187	2,977.4<br>2,237.6 to 4,171.5
Cellulitis	0.7<br>0.1 to 1.1	1,286<br>270 to 2,161	23.4<br>7.2 to 37.7	42,265<br>12,297 to 68,371	2,683.3<br>2,026.6 to 3,768.5
Pyoderma	0.2<br>0.0 to 0.4	437<br>63 to 839	8.5<br>2.5 to 14.7	15,340<br>4,304 to 26,728	5,780.7<br>4,276.5 to 8,683.5
Decubitus ulcer	0.0<br>0.0 to 0.1	67<br>14 to 115	5.9<br>4.1 to 8.0	10,784<br>7,530 to 14,837	1,581.2<br>1,109.2 to 2,248.2
Other skin and subcutaneous diseases	0.0<br>0.0 to 0.0	38<br>8 to 69	41.9<br>20.4 to 76.1	72,438<br>35,403 to 131,068	1,634.2<br>1,193.4 to 2,355.2
Sudden infant death syndrome	0.4<br>0.3 to 0.5	414<br>297 to 565	34.7<br>24.9 to 47.4	35,646<br>25,612 to 48,712	-33.8<br>-58.3 to 4.2
Injuries	103.0<br>72.6 to 139.7	174,721<br>123,196 to 237,982	5,950.9<br>4,494.5 to 7,748.4	9,566,861<br>7,199,469 to 12,563,169	-27.5<br>-44.9 to -4.9
Transport injuries	19.8<br>14.1 to 27.0	31,576<br>22,355 to 43,035	1,187.9<br>879.1 to 1,544.9	1,810,217<br>1,339,060 to 2,345,458	-25.1<br>-44.3 to -0.5
Road injuries	17.8<br>12.7 to 24.2	28,198<br>19,946 to 38,155	1,078.5<br>804.5 to 1,397.8	1,644,223<br>1,225,271 to 2,120,945	-25.2<br>-44.6 to -1.6
Pedestrian road injuries	5.7<br>4.0 to 7.9	9,632<br>6,837 to 13,296	297.1<br>219.2 to 402.3	464,853<br>341,588 to 627,684	-49.2<br>-66.2 to -25.1
Cyclist road injuries	0.4<br>0.3 to 0.6	730<br>500 to 1,034	53.7<br>39.9 to 69.5	89,329<br>65,752 to 116,834	-26.5<br>-47.7 to -0.7
Motorcyclist road injuries	1.2<br>0.8 to 1.7	1,787<br>1,180 to 2,523	86.4<br>62.5 to 115.9	125,785<br>91,604 to 167,470	-16.0<br>-44.8 to 29.8
Motor vehicle road injuries	10.3<br>7.2 to 14.2	15,906<br>10,882 to 22,036	628.6<br>461.1 to 837.3	942,893<br>689,477 to 1,252,947	-0.6<br>-28.0 to 39.7
Other road injuries	0.1<br>0.1 to 0.1	144<br>96 to 220	12.6<br>9.1 to 17.0	21,361<br>15,372 to 29,077	-43.4<br>-60.3 to -17.2
Other transport injuries	2.1<br>1.4 to 2.9	3,378<br>2,343 to 4,767	109.5<br>77.7 to 150.2	165,995<br>117,799 to 227,566	-24.2<br>-44.7 to 6.4
Unintentional injuries	37.8<br>27.4 to 52.5	66,483<br>47,724 to 93,400	2,642.9<br>2,062.3 to 3,384.9	4,394,584<br>3,409,972 to 5,670,803	-46.6<br>-59.1 to -31.1
Falls	7.4<br>5.3 to 10.0	13,622<br>9,803 to 18,232	903.7<br>677.9 to 1,173.5	1,591,635<br>1,197,314 to 2,067,549	-18.3<br>-38.8 to 5.7
Drowning	6.4<br>4.5 to 8.5	10,083<br>7,063 to 13,606	319.0<br>233.3 to 421.5	460,939<br>328,292 to 613,051	-65.9<br>-75.2 to -55.3
Fire, heat, and hot substances	4.7<br>3.3 to 6.3	8,429<br>5,972 to 11,391	251.8<br>194.0 to 322.6	415,319<br>315,189 to 537,251	-32.9<br>-50.1 to -8.4

Poisonings	1.5<br>1.1 to 2.2	2,351<br>1,639 to 3,593	84.0<br>62.8 to 112.0	118,267<br>86,918 to 166,260	-47.1<br>-61.5 to -29.4
Exposure to mechanical forces	3.0<br>2.1 to 4.2	4,875<br>3,375 to 6,905	273.4<br>208.3 to 359.8	440,327<br>334,627 to 577,452	-47.7<br>-63.4 to -24.7
Unintentional firearm injuries	0.5<br>0.3 to 0.7	785<br>542 to 1,107	23.5<br>16.7 to 33.0	37,080<br>26,374 to 51,339	-46.1<br>-59.8 to -23.2
Unintentional suffocation	0.6<br>0.4 to 0.9	775<br>550 to 1,205	43.2<br>31.4 to 68.7	56,766<br>41,757 to 85,727	-25.0<br>-43.0 to 2.5
Other exposure to mechanical forces	1.9<br>1.3 to 2.9	3,315<br>2,221 to 4,909	206.7<br>154.9 to 276.4	346,480<br>259,110 to 463,095	-51.6<br>-68.0 to -26.3
Adverse effects of medical treatment	1.2<br>0.8 to 1.7	2,193<br>1,515 to 3,112	45.5<br>32.1 to 62.2	74,917<br>52,477 to 103,314	13.0<br>-11.9 to 54.4
Animal contact	0.2<br>0.2 to 0.4	406<br>270 to 703	25.9<br>19.0 to 34.8	41,337<br>30,084 to 55,918	-24.5<br>-43.9 to -1.5
Venomous animal contact	0.1<br>0.1 to 0.2	241<br>152 to 435	17.8<br>12.6 to 24.2	28,029<br>19,693 to 38,158	-26.3<br>-46.4 to -1.9
Non-venomous animal contact	0.1<br>0.1 to 0.2	165<br>99 to 290	8.1<br>5.7 to 11.4	13,308<br>9,307 to 18,687	-21.6<br>-39.1 to 5.6
Foreign body	4.3<br>3.0 to 6.6	7,534<br>5,224 to 11,800	228.2<br>169.6 to 331.4	364,526<br>267,385 to 536,118	-31.6<br>-49.2 to -9.4
Pulmonary aspiration and foreign body in airway	4.0<br>2.8 to 6.3	7,140<br>4,945 to 11,258	204.4<br>151.9 to 303.5	326,227<br>238,313 to 490,925	-30.9<br>-48.8 to -8.8
Foreign body in other body part	0.2<br>0.2 to 0.4	394<br>269 to 674	21.2<br>15.3 to 29.6	34,170<br>24,677 to 48,094	-40.6<br>-56.8 to -13.6
Environmental heat and cold exposure	7.2<br>4.5 to 13.7	13,648<br>8,568 to 26,341	337.9<br>229.6 to 585.3	608,816<br>412,402 to 1,064,979	-56.7<br>-68.6 to -40.1
Other unintentional injuries	2.0<br>1.4 to 2.7	3,343<br>2,341 to 4,515	173.5<br>131.0 to 223.3	278,501<br>209,355 to 359,674	-45.1<br>-66.9 to -21.2
Self-harm and interpersonal violence	45.3<br>30.9 to 64.5	76,597<br>52,506 to 108,524	2,110.5<br>1,452.7 to 3,006.9	3,346,754<br>2,304,954 to 4,762,368	0.5<br>-27.2 to 34.2
Self-harm	30.6<br>20.6 to 43.6	52,001<br>35,189 to 75,088	1,363.4<br>904.8 to 1,934.0	2,160,200<br>1,447,926 to 3,076,816	-1.9<br>-30.7 to 32.4
Self-harm by firearm	1.1<br>0.6 to 1.8	1,923<br>1,104 to 3,106	47.8<br>26.8 to 75.6	77,446<br>43,926 to 123,440	-9.2<br>-39.9 to 28.9
Self-harm by other specified means	29.5<br>19.9 to 42.2	50,078<br>33,911 to 72,349	1,315.6<br>874.4 to 1,870.2	2,082,754<br>1,400,016 to 2,996,503	-1.6<br>-30.4 to 32.5
Interpersonal violence	14.7<br>8.4 to 23.5	24,596<br>14,019 to 39,655	747.1<br>464.8 to 1,154.9	1,186,550<br>730,943 to 1,847,659	6.4<br>-22.5 to 41.8
Physical violence by firearm	1.1<br>0.4 to 3.0	1,673<br>708 to 4,760	54.5<br>23.4 to 158.6	83,049<br>35,089 to 241,588	-9.9<br>-43.2 to 46.1
Physical violence by sharp object	5.1<br>2.7 to 8.8	8,462<br>4,522 to 14,450	252.9<br>139.0 to 427.7	397,101<br>217,899 to 676,978	-7.1<br>-36.3 to 25.6
Physical violence by other means	8.5<br>4.8 to 13.5	14,462<br>8,120 to 22,970	428.7<br>264.1 to 650.4	691,042<br>422,799 to 1,046,558	20.2<br>-14.4 to 64.9
Forces of nature, conflict and terrorism, and executions and police conflict	0.0<br>0.0 to 0.1	65<br>39 to 99	9.6<br>3.7 to 23.6	15,327<br>5,799 to 37,856	13.7<br>-32.9 to 80.0
Exposure to forces of nature	0.0<br>0.0 to 0.0	0<br>0 to 0	1.6<br>0.4 to 4.3	2,732<br>617 to 7,358	-

Conflict and terrorism	0.0<br>0.0 to 0.0	0<br>0 to 0	5.6<br>1.3 to 16.4	8,910<br>2,072 to 26,044	-
Executions and police conflict	0.0<br>0.0 to 0.1	65<br>39 to 99	2.5<br>1.5 to 3.7	3,685<br>2,225 to 5,580	13.7<br>-32.9 to 80.0

Appendix Table 2A. Percent of total deaths attributable to risks at Level 3 of the GBD hierarchy in 2016, for males age 15 to 49 years in Russia, China, Finland, Germany, Japan, Kazakhstan, Ukraine, and United States. Risks attributable to less than 1% of total deaths in Russia are not shown

	Russian Federation	China	Finland	Germany	Japan	Kazakhstan	Ukraine	United States
**Level 3 Risk Factor**	Percent 95% UI	Percent 95% UI	Percent 95% UI	Percent 95% UI	Percent 95% UI	Percent 95% UI	Percent 95% UI	Percent 95% UI
Alcohol use	34.4% (29.5-38.6)	14.9% (12.5-17.7)	30.9% (25.5-35.8)	20.0% (15.2-24.9)	10.3% (4.3-15.4)	25.4% (19.6-31.2)	33.1% (28.5-37.9)	18.2% (14.1-22.4)
Smoking	13.0% (11.4-14.6)	15.4% (11.7-20.3)	4.7% (3.6-5.8)	14.0% (11.6-16.0)	11.0% (9.2-12.9)	15.3% (13.4-17.1)	15.3% (13.2-17.3)	8.1% (7.0-9.0)
Drug use	10.6% (9.0-12.3)	3.0% (2.6-3.8)	14.5% (11.4-17.3)	7.4% (6.2-8.6)	5.2% (4.5-6.2)	6.1% (5.4-6.9)	9.9% (8.5-11.6)	15.9% (14.7-17.3)
High systolic blood pressure	9.0% (7.2-10.7)	12.5% (10.5-14.4)	7.3% (5.9-8.8)	8.9% (7.1-10.6)	10.7% (8.8-12.5)	12.4% (10.3-14.4)	13.3% (11.1-15.2)	7.5% (6.1-8.8)
High total cholesterol	7.9% (6.7-9.0)	7.7% (6.8-8.6)	5.0% (4.0-6.2)	8.2% (6.8-9.5)	6.8% (6.0-7.6)	10.1% (8.7-11.7)	10.4% (8.9-12.0)	7.2% (6.5-8.0)
High body-mass index	7.0% (4.5-9.3)	8.3% (4.2-12.9)	6.3% (4.0-8.5)	8.7% (5.4-11.6)	6.5% (3.5-9.6)	7.9% (4.5-11.3)	7.7% (4.6-10.8)	11.7% (8.9-14.0)
Diet low in whole grains	4.9% (3.4-6.6)	6.4% (4.5-8.7)	3.2% (2.1-4.4)	4.2% (2.8-5.9)	5.5% (3.8-7.2)	6.6% (4.6-8.6)	6.2% (4.3-8.4)	4.6% (3.2-6.0)
Diet low in fruits	3.7% (2.2-5.3)	6.3% (4.1-8.7)	2.4% (1.4-3.5)	3.7% (2.2-5.4)	5.8% (3.8-7.8)	5.3% (3.2-7.6)	5.2% (3.1-7.4)	3.0% (1.8-4.5)
Diet low in nuts and seeds	3.4% (2.1-4.6)	3.8% (2.5-5.1)	2.4% (1.6-3.4)	3.2% (1.9-4.6)	3.1% (2.0-4.3)	4.6% (2.9-6.4)	5.0% (3.2-6.7)	2.2% (1.2-3.3)
High fasting plasma glucose	3.3% (2.1-4.8)	5.1% (3.7-6.9)	2.7% (2.0-3.6)	3.0% (2.3-4.1)	3.5% (2.5-4.9)	3.0% (1.9-4.5)	2.4% (1.4-3.7)	4.7% (3.9-5.9)
Ambient particulate matter pollution	3.2% (2.5-4.0)	7.2% (6.1-8.3)	1.0% (0.6-1.5)	2.8% (2.0-3.8)	3.2% (2.4-3.9)	4.4% (3.5-5.4)	4.4% (3.4-5.4)	2.1% (1.5-2.7)
Unsafe sex	2.9% (1.7-4.5)	1.5% (1.3-1.6)	0.1% (0.1-0.1)	0.7% (0.6-0.9)	0.1% (0.1-0.1)	0.4% (0.3-0.6)	4.3% (2.9-6.2)	1.5% (1.5-1.6)
Diet low in vegetables	2.4% (1.1-4.0)	2.1% (0.8-3.7)	1.8% (0.9-2.9)	2.6% (1.2-4.2)	1.2% (0.3-2.5)	0.9% (0.0-2.7)	1.6% (0.3-3.4)	2.3% (1.0-3.7)
Diet low in legumes	2.1% (0.9-3.3)	2.0% (0.9-3.2)	1.4% (0.6-2.2)	2.3% (1.0-3.5)	0.4% (0.1-0.9)	3.3% (1.5-5.1)	2.8% (1.3-4.5)	1.8% (0.8-2.8)
Diet low in seafood omega-3 fatty acids	2.1% (0.9-3.5)	2.9% (1.3-4.5)	1.4% (0.6-2.4)	2.3% (1.0-3.8)	0.0% (0.0-0.1)	3.0% (1.2-5.0)	3.4% (1.4-5.5)	1.8% (0.7-3.0)
Diet low in fiber	1.7% (0.9-2.7)	2.0% (1.2-2.9)	1.2% (0.7-1.9)	2.0% (1.1-3.0)	1.6% (0.9-2.5)	2.4% (1.3-3.7)	2.3% (1.2-3.5)	1.4% (0.7-2.2)
Impaired kidney function	1.5% (1.2-1.9)	3.0% (2.6-3.5)	0.6% (0.5-0.8)	1.0% (0.8-1.2)	1.8% (1.6-2.1)	2.5% (2.1-3.0)	1.9% (1.5-2.4)	1.9% (1.7-2.1)
Diet high in sodium	1.4% (0.0-3.3)	5.8% (2.9-9.1)	1.2% (0.0-2.7)	1.6% (0.0-3.8)	3.4% (1.2-6.3)	3.1% (0.3-6.7)	2.0% (0.1-4.6)	2.3% (0.5-4.5)
Occupational carcinogens	1.0% (0.7-1.4)	2.3% (1.8-2.9)	0.8% (0.6-1.1)	1.9% (1.4-2.5)	2.1% (1.6-2.6)	1.8% (1.4-2.4)	1.1% (0.7-1.4)	1.3% (0.9-1.6)

Appendix Table 2b. Percent of total deaths attributable to risks at Level 3 of the GBD hierarchy in 2016, for females age 15 to 49 years in Russia, China, Finland, Germany, Japan, Kazakhstan, Ukraine, and United States. Risks attributable to less than 1% of total deaths in Russia are not shown

	Russian Federation	China	Finland	Germany	Japan	Kazakhstan	Ukraine	United States
**Level 3 Risk Factor**	Percent 95% UI	Percent 95% UI	Percent 95% UI	Percent 95% UI	Percent 95% UI	Percent 95% UI	Percent 95% UI	Percent 95% UI
Alcohol use	20.1% (17.1-23.4)	2.2% (1.6-2.9)	17.2% (13.7-20.7)	14.3% (11.3-17.4)	10.9% (6.4-14.9)	17.9% (13.9-22.3)	21.9% (17.7-26.1)	10.1% (7.8-12.6)
High body-mass index	8.1% (6.1-10.0)	8.2% (4.2-12.7)	5.5% (4.1-6.9)	6.1% (4.5-7.7)	2.9% (1.3-4.9)	11.5% (8.4-14.5)	8.9% (6.3-11.4)	10.9% (9.0-12.5)
Smoking	7.8% (6.5-9.3)	4.0% (1.8-7.8)	4.7% (3.5-6.0)	12.8% (10.8-15.0)	4.5% (3.8-5.4)	6.3% (4.8-7.9)	8.1% (6.3-9.9)	9.8% (8.9-10.9)
Drug use	7.7% (6.3-9.8)	2.1% (1.8-2.7)	7.0% (5.7-8.5)	3.7% (3.1-4.4)	2.9% (2.6-3.3)	3.9% (3.5-4.3)	9.1% (7.3-11.3)	12.4% (11.8-13.1)
High systolic blood pressure	7.6% (6.1-9.1)	9.9% (8.0-11.6)	5.2% (4.1-6.5)	4.9% (3.5-6.2)	5.0% (3.9-6.1)	10.1% (8.1-12.1)	10.0% (8.2-11.6)	5.3% (4.3-6.5)
Unsafe sex	6.3% (4.9-8.7)	4.5% (3.1-5.0)	1.1% (1.0-1.3)	3.4% (3.0-3.9)	4.2% (3.9-4.5)	4.2% (3.8-4.8)	8.9% (6.7-12.2)	3.3% (3.1-3.4)
High total cholesterol	5.7% (4.9-6.6)	5.9% (5.1-6.8)	2.2% (1.7-2.7)	3.6% (3.0-4.4)	2.9% (2.6-3.2)	7.2% (6.1-8.3)	7.4% (6.3-8.6)	4.9% (4.4-5.5)
Diet low in whole grains	4.7% (3.5-6.1)	5.9% (4.1-7.9)	3.0% (2.2-3.9)	3.1% (2.2-4.2)	3.4% (2.4-4.5)	6.4% (4.8-8.2)	5.6% (4.1-7.3)	4.2% (3.1-5.5)
Diet low in fruits	3.5% (2.3-5.0)	5.5% (3.4-7.6)	2.3% (1.5-3.4)	3.0% (1.8-4.2)	3.9% (2.6-5.2)	5.4% (3.5-7.3)	4.7% (2.9-6.5)	3.0% (1.9-4.2)
High fasting plasma glucose	2.8% (1.9-4.1)	5.2% (4.0-6.7)	2.6% (1.9-3.5)	2.5% (1.8-3.4)	2.6% (1.8-3.7)	3.4% (2.5-4.8)	2.8% (1.9-3.8)	5.7% (4.6-6.9)
Ambient particulate matter pollution	2.7% (2.2-3.4)	6.9% (5.8-8.0)	0.7% (0.5-1.1)	2.2% (1.6-2.7)	2.1% (1.6-2.6)	3.8% (3.1-4.6)	3.5% (2.8-4.3)	1.9% (1.4-2.4)
Diet low in nuts and seeds	2.4% (1.5-3.2)	3.1% (2.1-4.2)	1.2% (0.8-1.6)	1.5% (0.9-2.1)	1.3% (0.9-1.8)	3.2% (2.1-4.3)	3.6% (2.4-4.8)	1.8% (1.0-2.6)
Diet low in vegetables	2.2% (1.0-3.5)	2.1% (0.8-3.6)	1.4% (0.7-2.3)	1.8% (0.9-2.8)	0.9% (0.3-1.8)	1.0% (0.0-2.6)	1.5% (0.3-2.9)	1.9% (0.9-3.0)
Impaired kidney function	1.9% (1.6-2.2)	4.0% (3.5-4.6)	0.8% (0.6-1.0)	1.0% (0.9-1.2)	1.6% (1.4-1.9)	3.5% (3.1-4.0)	2.2% (1.8-2.6)	2.5% (2.3-2.7)
Diet low in seafood omega-3 fatty acids	1.5% (0.6-2.5)	2.3% (1.0-3.6)	0.6% (0.3-1.1)	1.1% (0.5-1.7)	0.0% (0.0-0.1)	2.1% (0.9-3.5)	2.4% (1.0-3.8)	1.3% (0.5-2.2)
Diet low in legumes	1.5% (0.7-2.3)	1.7% (0.8-2.7)	0.6% (0.3-1.0)	1.0% (0.5-1.6)	0.2% (0.1-0.5)	2.3% (1.0-3.5)	2.1% (0.9-3.3)	1.3% (0.6-2.1)
Diet low in fiber	1.5% (0.9-2.2)	1.9% (1.2-2.7)	0.9% (0.5-1.3)	1.3% (0.8-1.9)	1.1% (0.6-1.6)	2.0% (1.2-2.9)	2.0% (1.2-3.0)	1.2% (0.6-1.8)
Occupational carcinogens	1.2% (0.8-1.5)	1.9% (1.5-2.3)	1.0% (0.7-1.2)	1.9% (1.5-2.4)	1.7% (1.3-2.1)	1.8% (1.4-2.3)	1.2% (0.9-1.5)	1.7% (1.3-2.1)
Secondhand smoke	1.1% (0.8-1.5)	3.8% (2.9-4.7)	0.5% (0.3-0.7)	0.6% (0.4-0.8)	1.2% (0.9-1.5)	2.7% (2.1-3.4)	1.4% (1.0-1.8)	0.6% (0.4-0.7)
